# Stack: In-Context Learning of Single-Cell Biology

**DOI:** 10.64898/2026.01.09.698608

**Published:** 2026-01-09

**Authors:** Mingze Dong, Abhinav Adduri, Dhruv Gautam, Christopher Carpenter, Rohan Shah, Chiara Ricci-Tam, Yuval Kluger, Dave P. Burke, Yusuf H. Roohani

**Affiliations:** 1 Arc Institute; 2 Yale University; 3 University of California, Berkeley; 4 University of Pennsylvania

## Abstract

Single-cell transcriptomics offers the promise of measuring the diversity of cellular phenotypes across species, diseases, and other biological conditions. Recently, foundation models have emerged to identify this variation, yet most methods represent each cell independently, despite technical limitations that reduce measurement precision at the single-cell level. Here, we present Stack, a foundation model trained on 149 million uniformly preprocessed human single cells that leverages tabular attention to generate representations for each cell informed by the cells in its context. Stack offers substantial improvements for downstream tasks in the zero-shot setting compared to baselines, whether they are zero-shot, fine-tuned, or trained from scratch on the target dataset. Stack can perform in-context learning from unlabeled cells representing arbitrary conditions, such as a chemical perturbation or a different donor, and predict the effect of those conditions on a target cell population without requiring data-specific fine-tuning. We apply Stack to generate *Perturb Sapiens*, the first human whole-organism atlas of perturbed cells, spanning 28 tissues, 40 cell classes, and 201 perturbations. We validated subsets of *Perturb Sapiens* using *in vitro* stimulation profiles. Overall, Stack presents a new modeling framework where cells themselves act as guiding examples at inference time, unlocking general-purpose in-context learning capabilities for single-cell biology.

## 1. Introduction

The advent of large-scale single-cell RNA sequencing has generated unprecedented volumes of cellular data, with collections now exceeding hundreds of millions of cells across diverse tissues and conditions (Program et al., 2025; Youngblut et al., 2025; Consortium* et al., 2022). This data explosion has enabled the development of single-cell foundation models that leverage self-supervised learning to extract meaningful cellular representations from massive datasets (Theodoris et al., 2023; Cui et al., 2024; Rosen et al., 2023; Hao et al., 2024; Ding et al., 2025). These models are subsequently fine-tuned for various downstream tasks such as cell type annotation, batch integration, and perturbation effect prediction.

Despite their promise, current single-cell foundation models face significant limitations in their capacity for biological discovery. They often fail to surpass classical approaches when employed in a zero-shot manner (Kedzierska et al., 2025; Liu et al., 2023). Even with dataset-specific fine-tuning, they struggle to improve over simple baselines in perturbation prediction (Wu et al., 2024; Li et al., 2024; Kernfeld et al., 2023; Ahlmann-Eltze et al., 2025). Recent models (such as Adduri et al., 2025 and Klein et al., 2025) show improvements in these capabilities; however, they still require extensive training data and supervision on biological conditions and tasks of interest. This limits their potential for *de novo* biological discoveries, such as inferring perturbation effects in novel biological conditions (e.g. unseen cell types for which only observational data is available) or performing novel tasks, such as predicting sample-specific variation in immune phenotypes.

Most current single-cell foundation models are constrained by fundamental design choices. First, their pre-training objectives operate at the single-cell level, training models to function as universal “denoisers” that exploit gene dependencies but cannot see shifts at the population scale. Second, the inherently noisy count distribution of gene expression profiles necessitates aggregating information across cells to enhance signal-to-noise ratios of gene expression patterns. This insight has been employed in State for perturbation effect prediction (Adduri et al., 2025), but remains underexplored for single-cell foundation models. This aggregation is also essential for encoding inter-cellular interactions or mutual information that would otherwise be neglected or misattributed to gene-level dependencies. Third, prior models have largely been used for downstream tasks through task-specific fine-tuning on limited data, rather than exploiting the demonstrated ability of large transformer based models to perform robust learning at inference time.

To address these limitations and progress towards cellular foundation models that generalize beyond their supervised training conditions and tasks, we developed Stack, a self-supervised framework that enables in-context learning through a novel architecture on cell sets. Pre-trained on scBaseCount (Youngblut et al., 2025), the largest existing single-cell collection with 189 million high-quality human cells, Stack introduces several key innovations. The architecture employs customized transformer blocks that account for both intercellular and intra-cellular information flows, inspired by recent advances in tabular deep learning (Hollmann et al., 2025; Qu et al., 2025). A novel pre-training objective prevents simple memorization shortcuts while maintaining single-cell resolution. Additional improvements include enforcing linear identifiability in the latent space for better generalization, and a highly efficient dataloader for scalability.

After pre-training, Stack demonstrates the ability to automatically leverage cellular context information at the time of inference to refine embeddings and enable several downstream tasks, even for datasets never encountered during training. Through extensive evaluations, we show that this property enables substantial performance improvements in zero-shot cellular classification and integration tasks compared to various baseline models, whether they are zero-shot, fine-tuned, or even trained from scratch on the evaluation dataset.

Besides leveraging the context of a cell to enhance its embedding, we can also engineer the cell’s context to influence its state. Through a novel post-training alignment procedure, Stack introduces in-context learning (ICL) for single-cell foundation models. By post-training on an annotated 55-million-cell collection from CellxGene and the Parse peripheral blood mononuclear cell (PBMC) perturbation dataset (Program et al., 2025; Parse Biosciences, 2023), Stack learns to function as a conditional generative model analogous to masked diffusion models (Sahoo et al., 2024) at the cell dimension. The alignment enables “cell prompting” tasks, where Stack takes two cell populations termed *prompt* and *query*, and predicts how the query population would behave under the condition represented by the prompt. The framework empowers general tasks such as perturbation effect prediction and condition-specific cellular profile generation, supporting both perturbed and observational cells as prompts.

Our evaluation reveals that Stack can generate unseen context- or perturbation-specific cell types after post-training, on completely unseen data without data-specific fine-tuning. Across perturbation effect prediction and expression profile generation benchmarks, Stack’s zero-shot performance surpasses all evaluated strong baselines in 28 of 31 cases (Kernfeld et al., 2023; Adduri et al., 2025; Luecken et al., 2022). We applied Stack’s unique capacity to create the first perturbed human whole-organism atlas *Perturb Sapiens*, spanning 28 tissues and 40 cell classes under 201 drug and cytokine perturbations. *Perturb Sapiens* reveals realistic cellular responses across whole-organism cell types. The cell-type- and tissue-specific perturbation effects in *Perturb Sapiens* were validated using available *in vitro* cytokine stimulation datasets.

## 2. Results

### 2.1. Stack leverages cellular context to learn cell representations that generalize across datasets and tasks

**Figure 1 ∣:**
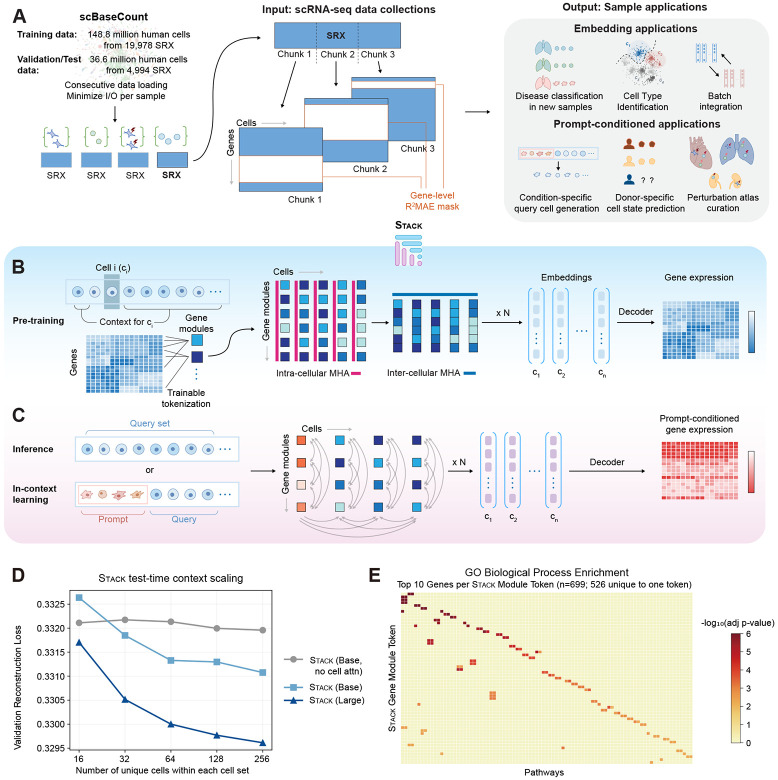
Stack: A single-cell foundation model that leverages cellular context. **A.** Overview of the Stack model. Stack takes scBaseCount human single-cell data (Youngblut et al., 2025) as input, comprising 149 million cells from 19,978 SRX files after preprocessing and filtering. Each file is chunked into consecutive cell sets of fixed size as model input. During pre-training, each input cell set is corrupted by masking a random subset of genes across all cells with a variable masking ratio (Dong et al., 2025). After pre-training, Stack enables zero-shot embedding analysis and a wide range of downstream tasks guided by designed prompts. **B.** Pre-training of Stack. Stack employs a single-layer multi-layer perceptron (MLP) to project cells into a set of gene module tokens. The tokens are passed through a tabular attention architecture that iteratively applies multi-head attention (MHA) along both gene and cell dimensions, followed by a feedforward network. After *N* tabular attention blocks, the final tokens are concatenated and flattened into one-dimensional vectors per cell (embeddings), and a decoder projects the embeddings back to expression space (Methods). **C.** Inference-time learning of Stack. The model takes either a single query cell set or a concatenation of prompt and query cell sets as input. Stack performs in-context learning through the tabular attention architecture, and can output prompt-conditioned gene expression. **D.** Effect of unique cell numbers on validation reconstruction loss under different Stack settings. All Stack models presented here were trained on the scBaseCount subset using identical training and validation sets (Methods). **E.** Heatmap of adjusted *p*-values from Gene Ontology (GO) biological process gene set enrichment analysis, showing the top 10 highest-importance genes (ranked by tokenization weight magnitude) for each Stack (Large) token after pre-training on full human scBaseCount. Module and pathway names, along with additional plot details, are provided in Fig. S2.

Stack is a large-scale self-supervised encoder-decoder model architecture designed to learn fundamental dependencies across cells and genes from single-cell data collections. We pre-trained Stack on human scBaseCount (Youngblut et al., 2025), the largest available human single-cell data collection that contains 189 million cells after strict quality control (Methods). The training dataset comprised 19,978 SRX samples and 149 million cells, with the remaining 20% of the data held out for validation and testing (Fig. 1A, Methods). Our high-quality training set is over four-fold larger than those of scGPT and Geneformer, and more than three-fold larger than that of UCE (Cui et al., 2024; Theodoris et al., 2023; Rosen et al., 2023). To accelerate model training, we developed a highly efficient h5py-based dataloader that reads consecutive chunks for each single-cell data sample and caches cell index sets (Fig. 1A, Methods). The dataloader achieves high input-pipeline throughput (around 1.6 × 10^4^ cells/second, over 75x faster than a similar model (Adduri et al., 2025)), sufficient to saturate GPU compute and complete pre-training in 2–3 days on a single H100 GPU.

The input to Stack is a collection of cells, or a *cell set*, from a single experimental sample. We define each cell’s *context* as the remaining cells in its set. Stack makes use of a rectangular mask pre-training task that prevents simple imputation shortcuts and enforces single-cell level resolution. Within a mini-batch, a randomly sampled list of genes are masked for all cells. The model is trained to reconstruct gene expression distributions for each individual cell. The mask ratio is sampled from a uniform distribution to enhance feature learning (Dong et al., 2025) (Fig. 1A). After pre-training, Stack outputs cell-level embeddings and gene expressions for new single-cell datasets, empowering numerous applications in both observational and perturbational biology in a zero-shot manner (Fig. 1A), without the need for test-data-specific fine-tuning. Stack gains strong zero-shot power through its inference-time learning capacity, which is detailed later.

Stack abstracts the latent state of each cell as an ensemble of token vectors, which we term “gene module tokens”. Tokens are generated by projecting gene expression vectors into a latent space using a single-layer perceptron, producing a fixed number of tokens per cell. This tokenization module is trained end-to-end alongside the rest of the model, without relying on external gene semantic information. To our knowledge, Stack represents the first single-cell foundation model to introduce trainable tokenization at the gene-group level. Because the number of tokens (100) is substantially smaller than the number of genes in the data, the model must implicitly learn meaningful gene groupings to preserve biological information. This also yields substantial scalability gains over gene-level tokenization.

A key innovation of Stack is a new tabular transformer block that enables both intra-cellular and intercellular information flow within the cell set (Fig. 1B). Each block stacks an intra-cellular multi-head attention (MHA) layer, an inter-cellular MHA layer, and a token-wise feedforward network (FFN) layer. In the intracellular MHA layer, the attention mechanism is performed on the gene module token sequence independently for each cell. In the inter-cellular MHA layer, the attention mechanism is on the cell set, with “cell tokens” defined as the concatenation of all gene module tokens. Our design draws inspiration from emerging tabular learning architectures such as TabPFN and TabICL (Hollmann et al., 2025; Qu et al., 2025) and additionally accounts for attention between different gene modules across cells. The final-layer gene module tokens are concatenated to form the cell embedding, and a cell-wise multilayer perceptron (MLP) decoder models the observed gene expression as a probabilistic function of this embedding. In addition to the masked gene reconstruction objective, Stack incorporates a distributional regularization that enforces cell embeddings to decompose into a per-cell-set constant plus standard normal distributed samples (Methods), matching the linear identifiability condition for non-linear latent variable models (Khemakhem et al., 2020; Dong et al., 2024).

During pre-training, this architecture captures the dependency between each cell and its context (the remainder of the cell set), enabling greater control and refinement of predicted single-cell expression. Following a post-training procedure, it also allows for the modification of a cell set of interest (*query* set) using an artificially designed set of *prompt* cells that implicitly encode auxiliary conditioning to guide the final model output. This output includes both embeddings and predicted expression values for each target cell (Fig. 1C). The framework offers two key advantages at inference: (1) The dependencies across cells in a set, encoded in intercellular attention layers during pre-training, generalize to unseen datasets to improve zero-shot performance without model updates; (2) It provides a backbone for in-context learning, enabling “cell prompt engineering” at inference. Specifically, the context can be altered to achieve desired outcomes for the query cells, such as shifting gene expressions to match a new donor or predicting the effects of perturbations. Cell prompts can be obtained from any single-cell observational or perturbational dataset, providing high-quality representations of diverse condition signals (such as disease state, genetic/chemical perturbations, donor variability, age) in a unified transcriptomic space. Simulating query data in the prompt context enables both generalization to new biological contexts (cell types, donors etc.) as well as novel predictive tasks (such as perturbation, age etc.) not encountered during training. Both rely solely on prompt-provided information at inference time.

For model evaluation, we also trained Stack on a version of the CELLxGENE collection (Program et al., 2025) that contains 73.7 million human cells, and a 60-million-cell subset of human scBaseCount in addition to the full scBaseCount. We observed a scaling behavior in terms of various validation metrics across configurations of Stack from 69 to 629 million parameters (Fig. S1A). Increasing hidden dimensionality results in an overall improvement of validation performance. Increasing network depth yields similar validation loss but improves performance on other metrics for the full scBaseCount dataset, while showing mixed effects on the scBaseCount subset (Fig. S1B). Scaling cell set size to 256 optimizes validation loss, while validation reconstruction metrics peak at a cell set size of 128 among the tested values (Fig. S1C). An ablation study confirmed that inter-cellular attention and latent space regularization in Stack both improve validation metrics (Fig. S1D). To assess the impact of informative cell context, we varied unique cells per cell set while holding total context size constant through repetition. Stack outperforms the ablation model without intercellular attention once unique cell numbers in the set exceed 32. The larger Stack model achieves lower validation loss, with gains widening as unique cells increase, indicating enhanced information aggregation capacity (Fig. 1D).

Finally, Stack tokenization yields highly specific gene groups. Among the top-10 genes per module (ranked by tokenization weight magnitude) , 526 of 699 (75.3%) appear in exactly one gene-module token, even though we impose no explicit sparsity objective. Gene set enrichment analysis confirmed that these tokens are functionally coherent (Fig. 1E, S2).

### 2.2. Stack generates superior embeddings by learning from cellular contexts at inference time

**Figure 2 ∣:**
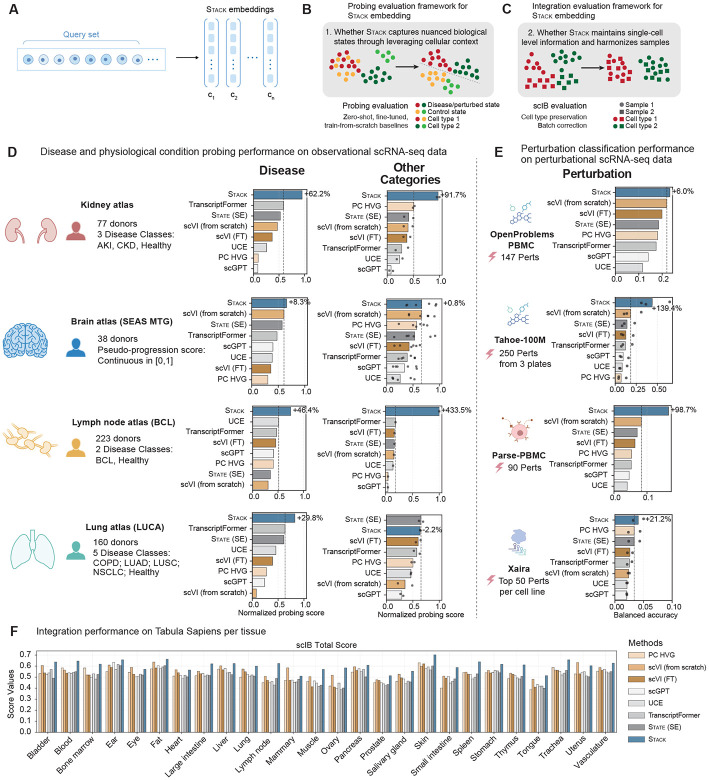
Evaluation of Stack embeddings. **A.** In the evaluations presented here, Stack serves as a context-aware embedding model, processing query cell sets from individual samples. **B.** Schematic illustration of the probing evaluation framework. **C.** Schematic illustration of the integration evaluation framework. **D.** Per-cell-type linear probing results. Probing scores represent the balanced accuracy (for classification tasks) or Pearson r (for regression tasks), which is first normalized for each cell type to a [0, 1] scale across all methods, and then averaged across all cell types. The number of experiments per dataset (equal to cell types): n=12, 20, 10, 20. AKI: Acute kidney injury. CKD: Chronic kidney disease. BCL: B-cell lymphoma. COPD: Chronic obstructive pulmonary disease. LUAD: Lung adenocarcinoma. LUSC: Lung squamous cell carcinoma. NSCLC: Non-small cell lung cancer. Other categories: Kidney (hypertension, diabetes history), Brain (Microinfarct pathology, ADNC, Braak stage, Thal phase, CERAD score, APOE4 status), Lymph node (LymphoMAP), LUCA (UICC stage, ever smoker). ADNC, Alzheimer’s Disease Neuropathologic Change. UICC, Union for International Cancer Control. **E.** Linear probing evaluation on classifying perturbations from 4 perturbation atlases. The number of experiments (evaluated cell types) per dataset: n=6, 60 (20 per plate), 17, 2. **F.** scIB evaluation for each tissue in Tabula Sapiens (Consortium* et al., 2022). All Stack results presented here are based on one model with the (Large) setting pre-trained on full human scBaseCount.

To evaluate the capabilities of Stack embeddings for individual samples on downstream tasks (Fig. 2A), we developed a comprehensive benchmarking framework that assesses the impact of cellular context and the quality of single-cell level representations, through probing and integration evaluations (Fig. 2B-C, Methods). The datasets for evaluation include: 1) five collections of observational samples, four representing distinct tissues (Kidney, Lymph Node, Brain, and Lung) drawn from a large number of donors (38–223), and Tabula Sapiens (De Boer et al., 2021; Li et al., 2025; Salcher et al., 2022; Gabitto et al., 2024; Consortium* et al., 2022), 2) four large-scale perturbation datasets covering three major perturbation modalities (Drug: OpenProblems, Tahoe-100M; Signaling: Parse-PBMC or Parse; Genetic: X-Atlas:Orion or Xaira) (Luecken et al., 2025; Zhang et al., 2025; Parse Biosciences, 2023; Huang et al., 2025). The LUCA dataset was part of the scBaseCount or CELLxGENE training data, whereas the remaining evaluation datasets were not, thus corresponding to a zero-shot setting (see Methods for details). We first evaluated model embeddings on observational single-cell data by applying linear and multi-layer perceptron (MLP) probes to predict metadata across varying levels of subtlety and resolution, ranging from disease and physiological conditions to cell types. Importantly, our probing schemes incorporate carefully designed procedures, including balanced donor cell numbers, donor-level test set holdout, and group-level cross-validation for regularization hyperparameter optimization (Methods). This rigorously tests whether the model captures biological state signatures through self-supervised learning that generalize to held-out donors.

We compared Stack with a list of methods that generate embeddings in zero-shot, fine-tuned, or train-from-scratch settings. These include principal components of highly variable genes (PC HVG), scGPT (Cui et al., 2024), UCE (Rosen et al., 2023), State (State Embedding or SE) (Adduri et al., 2025), TranscriptFormer (Pearce et al., 2025), scVI pre-trained on the scBaseCount subset and fine-tuned on the target dataset (scVI FT) (Lopez et al., 2018; Gayoso et al., 2022), and scVI trained from scratch on the target dataset (scVI from scratch). In per-cell-type linear probing, Stack shows a substantial advantage compared with alternative methods across disease/other categories on the four observational datasets (Fig. 2D, Fig. S3A). The only exception is the classification of other categories in LUCA, a dataset partially included in the training sets of both Stack and State (SE), where Stack ranks second and underperforms State (SE) by 2.2%. To control for the information flow across cell types, we constructed a new cellular context setting where the cells included in each cell set are constrained to be of the same cell type. Stack still achieves the best overall performance among all methods, albeit by a smaller margin (Fig. S4). Removing this cellular context information by shuffling the cell order results in a notable decrease in performance, with final results similar to those of the alternative methods (Fig. S4). To rule out the possibility that Stack ’s advantage arises from simple information leakage from more informative cell types, we further evaluated probing performance in the overall best-performing cell type across all methods, and Stack ’s advantage remained (Fig. S5). The association between Stack embedding performance and cellular context configurations suggests that Stack’s improvement stems from its ability to extract information from novel cellular contexts.

A similar advantage of Stack is observed with MLP probing across all four observational datasets (Fig. S3B). In either linear or MLP probing, all other advanced foundation models do not show consistent advantage over baseline methods such as PC HVG and scVI trained from scratch on the dataset of interest (Figs. 2D, S3, S4). In terms of cell type classification, all methods demonstrate highly similar performance (Fig. S3C). The small variation in performance is likely due to the cleaner cell type signal and its manually annotated nature. The competitive performance of Stack suggests that the context-aware mechanism does not compromise Stack’s representation at single-cell resolution.

We next evaluated different models’ performance in classifying chemical, signaling, and genetic perturbations (Fig. 2E). Stack outperforms existing methods on all perturbational datasets tested and is the only one to consistently outperform scVI trained from scratch. Notably, Stack shows substantial improvements in discriminating between perturbation effects in large-scale datasets Tahoe and Parse, outperforming the best alternatives by approximately 100% despite being trained almost exclusively on observational data. All methods show low absolute performance in classifying genetic perturbations in the Xaira dataset, likely due to measurement noise and gene expression similarity across perturbations. These results suggest that aggregating context information is crucial for accurate predictions of subtle biological states.

We also evaluated cell type preservation and donor label correction performance of different embeddings on observational data through scIB integration metrics (Luecken et al., 2022). Across all four observational datasets, Stack ranks as the top performer, surpassing the best alternative (State (SE)) by +1.8% (Fig. S6A). In Tabula Sapiens, Stack outperforms alternative methods in 21 of 25 tissues (ranking second in eye, heart, mammary and uterus, where scVI from scratch performs best), demonstrating superior performance across human tissues (Fig. 2F). This batch integration ability is emergent as it is not explicitly enforced during training. Scaling Stack training data and model size each lead to improvements in batch integration performance (Fig. S6B). UMAP visualization supports Stack’s effectiveness in clustering fine-grained cell states and integrating batch-level information (Fig. S6C). The results remain similar across model scales and extend to dataset label integration evaluations (Fig. S6D-E). These results establish Stack as a powerful embedding model that enhances zero-shot prediction and integration by leveraging cellular contexts.

### 2.3. Stack enables in-context learning of novel predictive tasks with cells after post-training

During pre-training, Stack is exposed to sets of cells from the same biological sample (such as donor or experimental condition), limiting the base model’s utility for tasks where the context is engineered by the user to produce a desired cell state. This limitation parallels the necessity of supervised fine-tuning (SFT) and reinforcement learning (RL) for adapting pre-trained large language models to follow user instructions (Wei et al., 2022; Longpre et al., 2023; Ouyang et al., 2022). To teach Stack to follow instructions, we define a cell conditioning task that involves two cell populations: prompt and query. The prompt cells specify the desired biological state or condition, while the query cells specify the cell type of interest. The objective is to predict counterfactual states of query cells, i.e., their gene expression profiles under the prompt condition, encompassing tasks such as generalizing perturbation effects to new cell types and across datasets. Here, prompt and query cells can come from different datasets, comprise non-overlapping cell types, and their annotations may be unavailable. Therefore, a supervised approach is not feasible for this task, and a foundation model with zero-shot capabilities is crucial. The context-awareness of Stack makes it particularly suitable for these tasks.

We developed a novel post-training recipe via self-distillation to adapt the Stack model for the task. This post-training process is closely related to masked language diffusion models (Sahoo et al., 2024), whose training objective is to recover masked tokens within input sequences with a masking ratio ranging from 0 to 1. In this procedure, each cell set from a single biological sample is grouped by type and partitioned into two subsets: *prompt* cells, which remain visible, and *target* cells, which are held out, analogous to unmasked and masked tokens in masked diffusion models. During post-training, the target cells are replaced with type-matched *query* cells drawn from a different biological sample. Stack is then post-trained to reconstruct the held-out target cells from query cells, conditioned on the prompt cells. Through this post-training procedure, Stack learns to predict counterfactual states for any cell population conditioned on the prompt cells, enabling conditional cell state generation.

**Figure 3 ∣:**
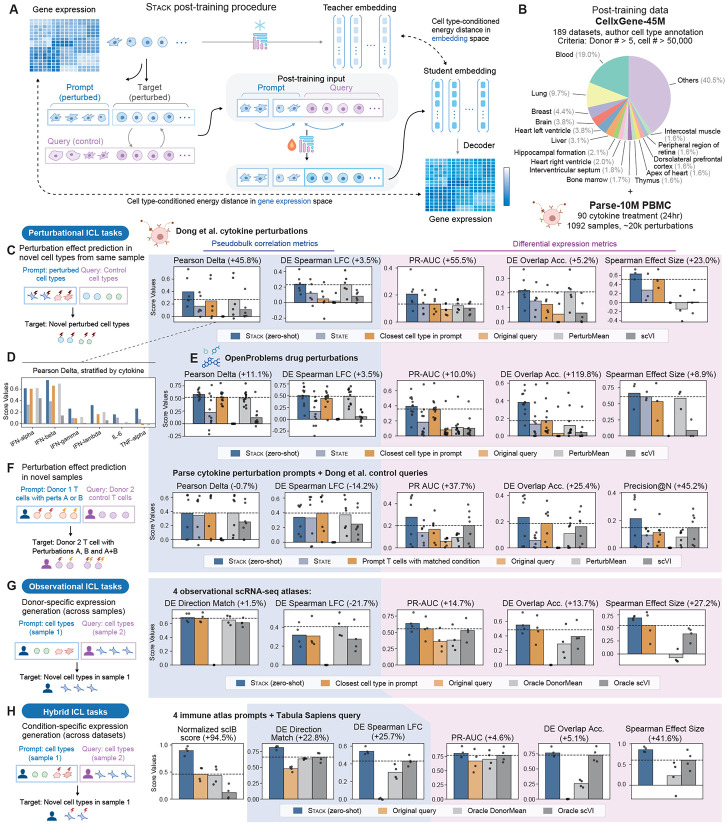
Post-training of Stack for in-context cell prompting tasks. **A.** Schematic illustration of the Stack posttraining framework. Cell sets are organized by cell type and partitioned into prompt and target cell sets. During posttraining, target cells are replaced with type-matched cells from different conditions (queries) to serve as model input. The model learns to predict gene expressions and embeddings of target cells via distributional alignment and self-distillation, with an MLP classifier guiding inference-time generation. **B.** Overview of post-training data. Training data comprised approximately 55M cells from curated CELLxGENE datasets and the Parse PBMC dataset (12 donors, 90 perturbations). **C.** Evaluation of perturbation effect prediction across cell types on the Dong et al. (2023) cytokine perturbation dataset (6 cytokines). In the first four panels, each point represents the average result of a cytokine condition. In the last panel, each point represents a cell type (B cells, myeloid cells, T cells). Percentages in titles represent the average improvement of Stack over the best non-Stack baseline. All Stack (zero-shot) predictions were generated using a post-trained Stack (Large) model with a mask diffusion procedure (*T* = 5). **D.** Pearson Delta evaluation results for Dong et al. (2023), stratified by cytokine. **E.** Evaluation of perturbation effect prediction across cell types on the OpenProblems drug perturbation dataset (Luecken et al., 2025) (12 drugs). Points represent drug conditions (first four panels) or cell types (last panel). **F.** Evaluation of T cell response prediction across samples. Each donor from Parse PBMC serves as a prompt, and donors a/b from (Dong et al., 2023) serve as queries. Each point represents the average result of a cytokine condition (7 single/combinatorial conditions). **G.** Evaluation of donor-specific gene expression generation across four atlases (Kidney: (De Boer et al., 2021); Lymph Node: (Li et al., 2025); Liver: (Edgar et al., 2025); PBMC: (Wells et al., 2025)). Nonoverlapping cell types from sampled donor pairs serve as prompts and queries. Each point represents one evaluation dataset. **H.** Evaluation of condition-specific expression generation across four PBMC atlases. Drug perts: 15 conditions with all PBMC cell type expression profiles available in (Luecken et al., 2025); Aging: first 10 donors in (Wells et al., 2025); Parse donors: all control PBS conditions from 12 donors; Parse perts: first 20 perturbation conditions from donor 1. Immune cells from Tabula Sapiens (Consortium* et al., 2022) serve as queries for all cases. Each point represents one evaluation case. For all scores, higher values indicate better performance.

We use a pre-trained Stack model as a teacher to extract embeddings for the target cells (Fig. 3A). The student Stack model is optimized to predict target cell distributions in both embedding and gene expression spaces, with additional regularization terms. The teacher model is updated using exponential moving average of the student model’s parameters, allowing it to progressively adapt to new data distributions while preserving knowledge acquired during pre-training. To compute the distributional match in gene expression space as a training objective, we employ a zero-inflated normal distribution approximation that enables reparameterization (see Methods). Finally, a multi-layer perceptron (MLP) classifier is trained to classify cells in the embedding space, where a lower score indicates greater similarity to the prompt condition and thus higher confidence in generation quality. At inference time, this score guides an iterative refinement procedure (Fig. 3A, Methods).

After post-training, we can use Stack as a conditional generative model to simulate novel cell populations. In the generative procedure, Stack receives cell sets containing concatenated prompt and query data. Stack predicts the gene expressions on all query positions, as well as their scores using the MLP classifier. At each iteration, we replace a fraction of highest-confidence query cells’ gene expression values with model predictions. This process is conducted in an iterative manner and finishes when the fraction reaches zero, at which point all query cells are replaced with predictions. Throughout the iteration, the fraction of prompt data in the input cell set is gradually increased to enable finer control (Methods).

For post-training data, we curated a large 55-million-cell scRNA-seq data collection comprising a set of large CELLxGENE datasets (>50,000 cells, >5 donors) and the Parse PBMC 10M dataset, which contains 12 donors and 90 cytokine perturbations (Fig. 3B). This training data emphasizes *in vivo* cell types, with particular focus on immune cells. To efficiently post-train on our large data collection, we developed an extended post-training dataloader that maximizes cell-type-aware local chunking. We evaluated the post-trained model on four downstream cell prompting/in-context learning tasks spanning three categories: perturbational, observational, and hybrid ICL tasks (Box 1).

Our evaluation leverages key metrics proposed in *cell-eval* (Adduri et al., 2025), which can be categorized into pseudo-bulk correlation metrics (Pearson Delta, DE Spearman LFC) and differential expression (DE) metrics (PR AUC, DE overlap accuracy, Spearman effect size; see Methods). We also report the Jaccard similarity metric, which measures the overlap between two DE gene sets normalized by their union, thereby adjusting for both predicted and ground-truth DE gene set sizes (Fig. S7). DE direction match and DE precision-at-N serve as auxiliary metrics when pseudobulk or DE metrics are less appropriate or not applicable (Methods). For setting 4, where target and query belong to different datasets, we additionally evaluated scIB batch integration metrics. Alternative baselines include the query data itself (input baseline), the nearest/same cell type in the prompt sample, State (Adduri et al., 2025), and two strong baselines identified in Adduri et al. (2025) (PerturbMean/DonorMean, scVI). For perturbational ICL tasks, we employ a “synthetic control” approach that uses the unperturbed version of the prompt sample to additionally predict a control profile, which then serves as the reference for the perturbation effect predictions when computing cell-eval metrics. The same synthetic-control procedure is applied to the closest/same-cell-type baseline and scVI, resulting in stronger baselines. For the remaining tasks, Stack operates without auxiliary samples, whereas DonorMean and scVI baselines still require them; we therefore designate these baselines as “oracle” in those cases. The evaluation data includes seven datasets that were **never seen** during either Stack pre-training or post-training: 1. OpenProblems drug perturbation (Luecken et al., 2025), 2. Cytokine stimulation (Dong et al., 2023), 3. Immune aging (Wells et al., 2025), 4. Tabula Sapiens (Consortium* et al., 2022), 5. Kidney atlas (De Boer et al., 2021), 6. Lymph node BCL (Li et al., 2025), 7. Liver atlas (Edgar et al., 2025). Additionally, we include the Parse PBMC dataset (Parse Biosciences, 2023) as prompts (not as queries) in our evaluations.

In setting 1 (perturbation effect prediction across cell types) for the Dong et al. (2023) cytokine stimulation dataset, Stack not only exhibits an advantage across all metrics (Fig. 3C, S7), but generalizes the global effect of subtle cytokine perturbations, including IL-6 and TNF-*α* across cell types, as demonstrated by Pearson Delta (Figs. 3D). The overall advantage also extends to the OpenProblems drug perturbation dataset, which comprises drug conditions unseen during model pre-training or post-training (Fig. 3E, S7). In setting 2, Stack outperforms alternative methods (including perturbation effects of observed prompt T cells) in DE metrics but performs similarly in pseudobulk metrics, across both individual and combinatorial cytokine conditions. Nevertheless, because Parse and Dong et al. (2023) use different stimulation protocols in dosage and stimulation duration, generalizing subtle effects of several cytokines (TNF-*α*, IL-6) alone yields near-zero pseudo-bulk and DE scores across all methods (Fig. 3F).

In setting 3, oracle DonorMean shows the strongest performance in pseudobulk correlation metrics, while Stack maintains its lead in directional and DE metrics across four evaluated tissues (Fig. 3G, S7). Notably, Stack demonstrates particular strength in generating cell types across datasets (Setting 4), as evidenced by scIB integration and cell-eval metrics (Figs. 3H, S7). This advantage may stem from the context-aware capacity acquired during Stack pre-training, as supported by the suboptimal performance of Stack trained from scratch on perturbational ICL tasks (Fig. S8).

As a zero-shot approach, Stack achieves the strongest overall performance across all ICL tasks, ranking first in 28 of 31 evaluations (Fig. 3C-H, S7). Closest/same-cell-type prompt cells emerges as a strong baseline when evaluated on cell-eval metrics, which has not been sufficiently addressed in previous perturbation prediction studies. Despite being trained on both prompt and query data, State is outperformed by Stack across all metrics on the evaluated perturbation tasks. This is likely because State was designed for settings with orders of magnitude more supervised data than those evaluated here. Stack’s success in low-data, cross-experiment settings demonstrates its utility as a foundation model when supervised fine-tuning is insufficient, for example, when interrogating cell types that are difficult to perturb experimentally. The multi-step mask diffusion generative procedure shows modest advantages over Stack one-step prediction and alternative generative schemes across ICL tasks (Fig. S9). The competitive performance of Stack on unseen prompts and queries (Dong et al., 2023; Wells et al., 2025; Consortium* et al., 2022), novel perturbations (Luecken et al., 2025), and cell types and tissues beyond peripheral blood (De Boer et al., 2021; Li et al., 2025; Edgar et al., 2025; Sikkema et al., 2023) underscores the generalizability of our approach to previously unencountered datasets and diverse biological tasks.

### 2.4. Stack generates a virtual whole-organism perturbational atlas

**Figure 4 ∣:**
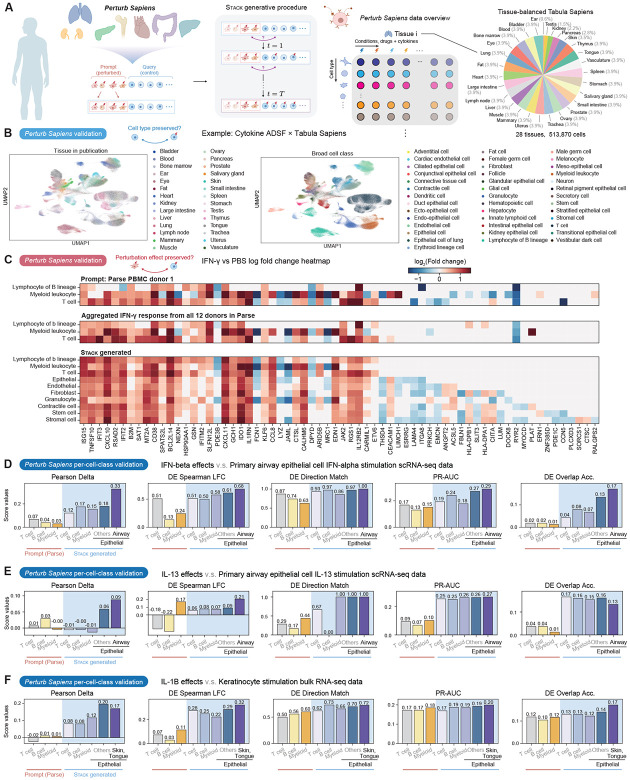
Analysis of a perturbational whole-organism atlas *Perturb Sapiens*. **A.** Overview of *Perturb Sapiens*. We utilize PBMC perturbation datasets (90 cytokines from Parse (Parse Biosciences, 2023); 144 drugs from OpenProblems (Luecken et al., 2025)) as prompts and Tabula Sapiens (Consortium* et al., 2022) as queries (left). For each tissue and cell type, *Perturb Sapiens* comprises simulated gene expression profiles under drug and cytokine perturbations. The resulting atlas spans 28 tissues and 513,870 cells per perturbation condition (right) (Consortium* et al., 2022). **B.** UMAP visualization of an example *Perturb Sapiens* generated by combining cytokine ADSF with Tabula Sapiens, colored by tissue and cell class. **C.** Log2-fold-change heatmap comparing IFN-*γ Perturb Sapiens* to control. Significantly differentially expressed genes are shown in color; non-significant genes are shown in gray. **D.** Evaluation of *Perturb Sapiens* epithelial interferonbeta (IFN-*β*) effects using single-cell IFN-*α* stimulation data from primary airway epithelial cells (Koh et al., 2023). **E.** Evaluation of *Perturb Sapiens* epithelial interleukin-13 (IL-13) effects using single-cell IL-13 stimulation data from primary airway epithelial cells (Koh et al., 2023). **F.** Evaluation of *Perturb Sapiens* epithelial interleukin-1 beta (IL-1*β*) effects using bulk IL-1*β* stimulation data from primary keratinocytes (Swindell et al., 2018).

We employed Stack to generate a perturbational whole-organism atlas (*Perturb Sapiens*) through ICL, using 90 cytokine perturbations in Parse and 111 drug perturbations in OpenProblems as the prompt and the entire profile of Tabula Sapiens, after tissue balancing, as the query (Fig. 4A) (Parse Biosciences, 2023; Luecken et al., 2025; Consortium* et al., 2022). UMAP visualization demonstrates a single-cell resolution map with tissue-specific and cell-type-specific expression for *Perturb Sapiens* (Figs. 4B, S10A). Inspection of the MLP classifier scores suggests a variation in generation confidence for different cell and tissue types (Figs. S10B-C). The classifier assigns low confidence (high logit value) to several rare cell types such as transitional epithelial cells in both drug and cytokine example perturbations. We restricted our subsequent analyses to cells with logits smaller than a threshold (2.5) suggesting high confidence.

As a representative example, we inspected the effect of IFN-*γ* perturbation versus control in *Perturb Sapiens* to assess model generalization beyond the effects observed in the prompt or prompt-related (immune) cell types. Stack generates a highly realistic differential expression map with cell-type specificity (Fig. 4C). The top differentially expressed genes exhibit near-perfect concordance between prompt and generated immune cells. Notably, although only a single donor from Parse was used as the prompt, *Perturb Sapiens* achieved stronger alignment with the aggregated response across all 12 Parse donors, demonstrating its capacity to overcome individual experimental noise (Fig. 4C). Known downstream targets of IFN-*γ* were activated across a variety of immune and non-immune cell types (e.g., *IFIT3, ISG15, CXCL10, CXCL11, IDO1*). In non-immune cell populations, IFN-*γ* induced *CIITA* in stromal and contractile populations while broadly suppressing extracellular matrix and adhesion genes (*LUM, FBLN1, LAMA4, ITGA8*); this was accompanied by vascular remodeling signatures (*EMCN, ANGPT2, CEACAM1, PLAT*) consistent with IFN-driven inflammatory reprogramming. These genes are either not differentially expressed in the Parse PBMC data or exhibit inconsistent expression trends between the prompt donor and the aggregated profile. This indicates that Stack successfully generalized beyond the prompt data to capture immunomodulatory and remodeling responses specific to non-immune lineages. Dactolisib perturbation leads to global repression of interferon-stimulated genes (ISGs) (Fig. S11), aligning with the known role of dactolisib as a PI3K/mTOR inhibitor and previous analysis (Dong et al., 2024). Additionally, non-immune lineages showed selective remodeling, with cytoskeletal/adhesion and stress-response programs being modulated (e.g., *TUBA1C, FHL1, LAMA2, NINJ1, MT1X*).

While a majority of drug perturbations in OpenProblems only contains the T cell lineage, Stack also generates high-quality predictions across all cell types as observed for proscillaridin-A and ketoconazole (Fig. S12-S13). Proscillaridin-A elicited a broad pro-inflammatory, innate-like activation program across both immune and non-immune cell types. Non-immune lineages exhibited distinct transcriptional remodeling involving vesicular trafficking and membrane dynamics (*RAB30, STX3, LYST*) alongside lineage-specific regulators (*CPEB4, PLAGL1, TSC22D1*). Notably, ketoconazole exhibited pronounced donor-specific effects across the OpenProblems dataset. *Perturb Sapiens* effectively captures these individualized responses in the prompt donor while maintaining alignment with bulk expression patterns for a number of genes where donor-specific differential expression is not detected (Fig. S13). Overall, top DEGs in drug perturbation conditions show strong concordance between prompt and generated immune cells comparable to the cytokine case, although the drug *Perturb Sapiens* yields additional positives where prompt immune cells also express predicted non-immune DEGs. For quantitative validation, we benchmarked *Perturb Sapiens* immune cells against biological replicates from the same drug/perturbation datasets, assessing their ability to capture prompt perturbation effects. Although *Perturb Sapiens* yields low Pearson Delta scores likely due to batch effects, it achieves superior DE overlap accuracy compared to biological replicates, and the performance of *Perturb Sapiens* remains consistent across both drug and cytokine perturbations (Fig. S14). These findings support Stack’s ability to generate biologically meaningful, cell-type-specific responses to diverse perturbations, without observing any perturbations in the query cell type.

We next quantitatively evaluated Stack’s performance on generating perturbed non-immune cells, focusing on the epithelial lineage due to the prevalence of *in vitro* epithelial cytokine stimulation datasets (Koh et al., 2023; Lee et al., 2022; Swindell et al., 2018; Saito et al., 2021). We benchmarked the model’s ability to reproduce the effects of five cytokines (type I IFN, IL-13, IL-1*β*, TNF-*α*, IL-17A) in single-cell or bulk data using either direct prediction of each cytokine or functionally similar cytokines, applying the same set of cell-eval pseudobulk and DE metrics as before. Stack demonstrated strong performance in generating epithelial-specific type I IFN responses, outperforming both generated immune cell types and Parse immune cells used as the prompt (Fig. 4D). Notably, the model exhibited a clear preference for airway epithelial cells over other epithelial subtypes, consistent with the *in vitro* experiment. Unlike IFN-*β*, IL-13 is a cytokine that primarily affects epithelial cells and has weaker effects on immune cells. Despite this limited signal in the prompt, *Perturb Sapiens* demonstrates cell-type-specific alignment with the *in vitro* airway epithelial experiment, albeit with overall lower scores (Fig. 4E). This cell-type specificity generalizes to other tissues, as observed in the IL-1*β* keratinocyte stimulation experiment (Fig. 4F). Across all three cases, *Perturb Sapiens* achieves better performance than the prompt data, with scores ordered by cell-type similarity. Evaluations without confidence filtering results in reduced cell-type specificity, confirming the essence of the procedure (Fig. S15).

For IL-17 and TNF-*α*, both the Parse prompt and generated data showed negative association with epithelial response (Fig. S16), with latter replicated in an independent TNF-*α* epithelial stimulation dataset (Table S1). Upon further investigation, we found that despite concordance in several TNF-*α* response genes, including metallothioneins and cell adhesion molecules, TNF-*α* suppressed the expression of genes involved in NF-*κ*B signaling and type I ISG programs in both Parse T cells and *Perturb Sapiens*, while inducing these signatures in the *in vitro* epithelial dataset (Table S2). This phenomenon aligns with previously documented secondary signaling effects of TNF-*α* (Kalliolias and Ivashkiv, 2016). In summary, our results suggest that Stack can simulate perturbed non-immune cells with cell-type and tissue specificity, with alignment to ground truth data correlating with the perturbation’s effect size and specific biological mechanisms.

Finally, we computed log-fold-changes and statistical significance of perturbation effects across all available conditions in *Perturb Sapiens*, stratified by cell type and tissue. This framework enables multi-scale characterization of perturbation responses and provides a unified approach to categorizing drug and cytokine perturbations. At the global scale, predicted perturbation similarities clustered coherently by cell lineage and tissue proximity (Fig. S17). At the local scale, we decomposed the concatenated perturbation effect space for each cell type and tissue using independent component analysis, revealing diverse response modules that segregated by perturbation type without evident batch effects across drugs and cytokines (Fig. S18A-C). Examination of the top contributing genes within each independent component identified IFN/inflammatory signaling as the dominant response axis, with additional modules reflecting stress response and ECM remodeling pathways (Fig. S18D-F). Collectively, *Perturb Sapiens* provides a comprehensive, multi-resolution view of perturbation effects across cell types and tissues, representing a rich resource that extends well beyond the analyses presented here.

## 3. Discussion

As large atlases of single-cell transcriptomic profiles are compiled across tissues, species, and diseases, foundation models present an exciting opportunity to learn universal biological principles and patterns that generalize beyond experimentally observed data (Bommasani et al., 2021). A virtual atlas of cell states could significantly expand our understanding of cell biology through uncovering cellular states that are difficult to probe experimentally but can be inferred through relationships learned from existing data (Bunne et al., 2024; Roohani et al., 2025). However, realizing this promise requires models that transfer robustly across conditions and tasks. Most existing models present several limitations: they often fail to generalize to previously unseen conditions, provide no benefit over methods that are specifically fine-tuned on those datasets, and cannot perform new tasks without being explicitly trained to do so.

Here, we introduce Stack, a single-cell foundation model that leverages information from the cellular context of each cell to create enhanced representations. This design enables Stack to consistently outperform models trained from scratch on each evaluation dataset, an outcome that, to our knowledge, has not been observed for any existing single-cell foundation model, and highlights Stack ’s ability to meaningfully lever-age information acquired during pre-training. This dependence on context also enables a novel capability: engineering context to design cell state. Because context can be defined in many ways, such as an applied perturbation, a disease state, or a new donor, Stack supports inference-time learning of new tasks, including zero-shot generalization to new biological contexts and datasets.

Through this capability of in-context learning, Stack enables a new approach to single-cell modeling in which counterfactual cell states can be generated via prompting with only cells. Importantly, Stack extends existing perturbation modeling by removing the reliance on perturbation labels, cell type encodings, and test-sample-specific controls, enabling direct, label-free comparison of cell states to resolve context-dependent responses that transcend categorical annotations. These results position Stack as a generative cellular model capable of predicting unobserved gene expressions across cell types, perturbations, and novel donors, with potential to accelerate therapeutic and drug discovery cycles. As a concrete demonstration, we applied Stack to generate *Perturb Sapiens*, an organism-wide atlas of perturbed cells spanning 28 tissues, 40 cell classes, and 201 perturbations, a resource we believe will be of broad value to the community.

Despite its strong performance and novel capabilities, Stack has several limitations that present opportunities for future research. The current model is trained exclusively on human single-cell data. Extending this framework to multi-species applications would require additional design in the tokenization procedure to account for gene misalignment across species. Model calibration for rare cell types and weak perturbation effects remains to be established, marking an important area for future development. Signaling cascades may introduce time-dependent or secondary perturbation effects as we observed for TNF-*α*, which may complicate interpretation of Stack predictions. Finally, as the model is post-trained primarily on *in vivo* cell types, especially immune cells, a more sophisticated data curation and alignment scheme may improve model generalization across *in vitro* perturbation studies and *in vivo* observational data.

## 4. Methods

### 4.1. Stack model

#### 4.1.1. Generative process

Stack models the state of cell k, $E^{\left(k\right)}\in R^{n\times d}$, as a ensemble of *n* token vectors $e_{i}^{\left(k\right)}\in R^{d}$, which we term *gene module* tokens, since each token represents a coherent subset of gene variability in the single cell:

(4.1) $$E^{\left(k\right)}=[e_{1}^{\left(k\right)};e_{2}^{\left(k\right)};\cdots ;e_{n}^{\left(k\right)}].$$

The flattened state vector ${\bar{E}}^{\left(k\right)}\in R^{nd}$ is linked to the ground truth gene expression $x^{\left(k\right)}\in R^{G}$ with the generative process considered in scVI models (Lopez et al., 2018; Gayoso et al., 2022). The generative process involves a latent decoding transformation f and cell library size scalar $l^{\left(k\right)}\in R$. The final expression count is modeled through a negative binomial (NB) distribution with mean and dispersion parameters ($\rho^{\left(k\right)},\theta^{\left(k\right)}$):

(4.2) $$(\rho^{\left(k\right)},\theta^{\left(k\right)})≔f({\bar{E}}^{\left(k\right)})\in (R^{G},R^{G});\;x_{g}^{\left(k\right)}∼\text{NB}(l^{k}\;\rho_{g}^{\left(k\right)},\theta_{g}^{\left(k\right)}).$$

This generative process may be seen as a hybrid of transformer-based data modeling and biophysical-like models considered in scVI. The most prevalent differences from both strategies are summarized as follows.

- Stack does not consider low-dimensional latent variables as in scVI models; the total cell token dimensionality *nd* ~ 10^3^, which is comparable to large language models and large-scale single-cell self-supervised-learning models (Cui et al., 2024; Rosen et al., 2023; Adduri et al., 2025).
- Stack does not include structural tokens (e.g. CLS tokens) as in several classic (single-cell) transformer models (Devlin et al., 2019; He et al., 2022; Rosen et al., 2023; Adduri et al., 2025). For downstream fine-tuning or linear probing on embeddings, the concatenation of all output tokens are used as the Stack model embedding output.

The generative process corresponds to the model decoding procedure from cell state embedding to gene expression, operating independently across cells.

#### 4.1.2. Stack architecture

During pre-training, the Stack model receives a cell set $X\in R^{K\times G}$, where K denotes the number of cells and G the total number of genes. The cell set includes cells continuously indexed from the same SRX experiment (for scBaseCount (Youngblut et al., 2025)) or the same dataset (for CELLxGENE (Program et al., 2025), where cells in datasets are primarily sorted by donor ID). The organization of each cell set introduces dependency across cells within the cell set, which serves as rich auxiliary information. Stack encodes the cell set $X\in R^{K\times G}$ with the following architecture:

- **Tokenization.** First, each cell k in the cell set $x^{\left(k\right)}\in R^{G}$ is projected into dimension n×d with a 1-layer perceptron, where *n* is the number of gene module tokens and *d* denotes the token size. Then a gene token embedding $P\in R^{n\times d}$ is added on the perceptron output. This results in a tensor $Z_{0}^{\left(k\right)}\in R^{K\times n\times d}$ for the cell set.
- **Tabular transformer layer.** The tensors are processed by a stack of $N_{L}$ tabular transformer layers (${\left\{{𝒯}_{i}\right\}}_{i=1}^{N_{L}}$):

(4.3) $$Z_{i}={𝒯}_{i}(Z_{i-1}),\;i\in \{1,2,\cdots ,N_{L}-1\};\;\;E={𝒯}_{N_{L}}(Z_{N_{L}}-1).$$

Each layer ${𝒯}_{i}$ applies a dual attention mechanism on the cell-set-level representations. In the following description, each attention module is a standard multi-head attention (MHA) block: the input is projected into multiple heads, attention outputs are concatenated and projected back, then combined with the input through a residual connection followed by layer normalization.

1. **Intra-cellular attention.** An intra-cell MHA module operates across the *n* tokens independently for each cell in $Z_{i}\in R^{K\times n\times d}$. The sequence length is *n*, and the feature dimension is *d*. The attention head number is $N_{H_{g}}$.
2. **Inter-cellular attention.** An inter-cell MHA module operates across K cells, with the (*n, d*) dimensions flattened into *nd*. The sequence length is K, and the feature dimension is *nd*. The attention head number is $N_{H_{c}}$.
3. **Feedforward network (FFN).** A position-wise FFN independently processes each *d*-dimensional token, followed by residual connection and layer normalization, yielding $Z_{i}\in R^{K\times n\times d}$.

- **Cell-wise decoder.** Finally, the cell state embedding $E\in R^{K\times n\times d}$ is decoded into gene expression space independently for each cell in {1,2,⋯,K}. The decoder f is implemented as a multi-layer perceptron (MLP) that maps the flattened embedding ${\bar{E}}^{\left(k\right)}\in R^{nd}$ the negative binomial parameters $\left(\rho^{\left(k\right)},\theta^{\left(k\right)})\in (R^{G},R^{G}\right)$, following the generative process described above.

#### 4.1.3. Pre-training objective

The model is pre-trained on a masked gene reconstruction task. For each input cell set X, a random subset of genes ℳ⊂{1,2,⋯,G} is selected, and their expression values are masked across all K cells. The masking ratio is randomly sampled from $\left(p_{\text{min}},p_{\max})=(0.1,0.8\right)$ for each mini-batch. This selection range aims to cover gene dependencies of various strengths, following R^2^MAE (Dong et al., 2025). We adopt a higher $p_{\max}$ (0.8) than in (Dong et al., 2025) (0.5) as the inter-cellular information brings additional information for implicit denoising. The pre-training objective combines a reconstruction loss with a latent space regularization term:

(4.4) $$ℒ={ℒ}_{\text{recon}}+\lambda_{\text{SW}}{ℒ}_{\text{SW}}.$$

The reconstruction loss, ${ℒ}_{\text{recon}}$, is the negative log-likelihood of the original counts under the predicted NB distribution, computed for the masked genes across all cells in the cell set X:

(4.5) $${ℒ}_{\text{recon}}=-\frac{1}{K|ℳ|}\sum_{k=1}^{K}\sum_{g\in ℳ}\log\;P(x_{g}^{\left(k\right)}|l^{\left(k\right)},\rho_{g}^{\left(k\right)},\theta_{g}^{\left(k\right)}).$$

Optimizing solely the reconstruction loss leads to memorization and a reduction in performance. To enforce meaningful pre-training, we incorporate a regularization term, ${ℒ}_{\text{SW}}$, defined as the Sliced Wasserstein distance between the empirical distribution of the final flattened cell states ${\left\{{\bar{E}}^{\left(k\right)}\right\}}_{k=1}^{K}$ and a batch-centered, multivariate Gaussian prior $𝒩(\frac{1}{K}\sum_{k=1}^{K}{\bar{E}}^{\left(k\right)},I_{nd})$. This term enforces the embedding to be decomposed into a centralized Gaussian distribution and a cell-set-specific constant vector, which regularizes embedding distribution and enforces linear identifiability of latent factors (Dong et al., 2024). The linear identifiability result follows directly from the theoretical framework established in Khemakhem et al. (2020). The loss term is calculated within a random cell set subset, with subset size uniformly sampled from [32, 33, · · · , 128]. The hyperparameter $\lambda_{\text{SW}}$ (default 0.01) balances the two loss components.

### 4.2. Post-training

The pre-trained Stack model is post-trained for in-context prompting tasks through a supervised alignment procedure. The objective is to empower the model to generate novel cell populations, combining cell type information from a set of *query* cells, and the biological context provided by a separate set of *prompt* cells. Our approach comprises four key components: (i) a novel self-distillation procedure, (ii) a training input construction strategy, (iii) architectural modifications, and (iv) a composite loss function that balances generalization with knowledge retention.

#### 4.2.1. Self-distillation

We introduce a self-distillation procedure during post-training. A frozen teacher model, operating without input data masking, computes embeddings and gene expression parameters from cell sets containing individual biological samples; these outputs are then used to calculate the training objectives. The teacher model weights are updated via exponential moving average (EMA) of the student model which is actively post-trained. This approach follows the self-distillation framework established in self-supervised vision models (Grill et al., 2020; Oquab et al., 2023; Zhou et al., 2021).

#### 4.2.2. Post-training input construction

Different from pre-training, the post-training process involves two biological samples with matched cell type annotations. We begin with a cell set of K cells $X={\left\{x^{\left(k\right)}\right\}}_{k=1}^{K}$ drawn from the prompt sample. Cells are ordered such that cells of the same type appear consecutively, with cell type order randomized for each sample. This cell set is partitioned into three components:

1. **Prompt condition cells** ($X_{\text{prompt}}^{\text{fixed}}$): The first 25% of cells, used unchanged as conditioning context.
2. **Prompt context cells** (${\hat{X}}_{\text{prompt}}^{\text{kept}}$): $K_{\text{kept}}$ cells whose expression profiles are sampled from the means and dispersions predicted by a teacher Stack model for the corresponding original cells.
3. **Target cell positions**: The remaining $K_{\text{query}}$ positions, where $K_{\text{kept}}+K_{\text{query}}=0.75K$ and the ratio $K_{\text{kept}}∕(K_{\text{kept}}+K_{\text{query}})∼𝒰(0,1)$.

The target cell positions are filled with *query cells* ($X_{\text{query}}$) drawn from a different biological context (e.g., a different donor or perturbation condition). Each query cell is matched by cell type to the target cell it replaces. This yields the final input:

(4.6) $$X_{\text{in}}=[X_{\text{prompt}}^{\text{fixed}},{\hat{X}}_{\text{prompt}}^{\text{kept}},X_{\text{query}}].$$

The model’s objective is to predict the gene expression distribution of target cells, conditioning on the prompt cells as reference. To ensure the model learns meaningful biological transitions, each replaced cell must have the same cellular identity (e.g., cell type or cell line, specified by user) as the original cell it replaces. To address imbalanced cell type distributions, we apply a balancing procedure to each training sample: overrepresented cell types are downsampled to the average count per type, then the resulting pool is upsampled with replacement to restore the original cell set size. For replicated cells, only the first instance contributes to the distributional loss terms which will be detailed in Section 4.2.4.

#### 4.2.3. Architectural modifications

We introduce two learnable modules to the model: a query position embedding, $P_{\text{query}}\in R^{nd}$ and an MLP binary classifier $f_{\text{CLS}}\;:R^{2nd}\to R$. The position embedding $P_{\text{query}}$ is added to the token representations of query cells at the first embedding layer. The MLP binary classifier $f_{\text{CLS}}\;:R^{2nd}\to R$ that takes the concatenation of mean prompt condition embedding and prompt context/query single-cell embedding as input, and predicts whether the cell comes from prompt (0) or query (1). The classifier receives detached embeddings as input, therefore its optimization is independent from other model weights. We register gradient hooks on these newly introduced modules to apply a 10× gradient scaling, which effectively increases their learning rates relative to the pre-trained parameters. Additionally, a causal attention mask is applied within all transformer layers to prevent prompt condition cells from attending to prompt context or query cells, ensuring that information flows strictly from prompt condition cells to the remaining cells.

#### 4.2.4. Post-training objective

Similar to the pre-training setup, the model receives a masked version of the input cell set $X_{\text{in}}$ with rectangular gene masks, with masking ratio sampled from 𝒰(0.1,0.3). The post-training objective is designed to simultaneously predict unseen target cells and maintain information learned during pre-training:

(4.7) $${ℒ}_{\text{FT}}={ℒ}_{\text{dist}}+\lambda_{\text{recon}}{ℒ}_{\text{recon}}+\lambda_{\text{SW}}{ℒ}_{\text{SW}}+\lambda_{\text{CLS}}{ℒ}_{\text{CLS}};\;{ℒ}_{\text{dist}}=0.5\times ({ℒ}_{\text{gene}}+{ℒ}_{\text{embed}}).$$

The components are defined as follows:

- **Embedding Alignment Loss (${ℒ}_{\mathrm{embed}}$):** The term is defined as the energy distance between Stack embeddings of query cells and target cells. The embedding for the target cells is extracted from the teacher model and is detached for loss calculation. The student model to be fine-tuned calculates the embedding for query cells from input data cell set $X_{\text{in}}$.
- **Expression Alignment Loss (${ℒ}_{\mathrm{gene}}$):** The term is defined as the energy distance between the predicted and true distributions of log normalized target gene expression. To make the optimization on NB distribution parameters tractable, predictions are generated using a reparameterizable zero-inflated normal distribution sampler that matches first two moments of the log-normalized NB distribution. To address over-smoothing, we estimate a shared over-dispersion parameter for query cells using the median detached over-dispersion computed from prompt cells. The loss is computed on first 1,000 highly variable genes per mini-batch (identified via Pearson residuals (Lause et al., 2021)), and is stratified by cell type like ${ℒ}_{\text{embed}}$.
- **Distributional Alignment Loss (${ℒ}_{\mathrm{dist}}$):** This is the primary alignment objective, which is defined as the average of embedding alignment loss ${ℒ}_{\text{embed}}$ and gene expression alignment loss ${ℒ}_{\text{gene}}$.
- **Reconstruction Loss (${ℒ}_{\mathrm{recon}}$):** To retain the model’s mask reconstruction capabilities, we apply a standard masked gene reconstruction loss, as in pre-training, to the prompt cells. This serves as an auxiliary task that regularizes the model and enforce it to maintain pre-training objective. We use $\lambda_{\text{recon}}=1$.
- **Latent Regularization (${ℒ}_{\mathrm{SW}}$):** The Sliced Wasserstein distance objective from pre-training is retained and applied to the embeddings of all cells in the batch. We use $\lambda_{\mathrm{SW}}=0.01$.
- **Classification Loss (${ℒ}_{\mathrm{CLS}}$):** The classification loss is defined as the binary cross entropy loss (BCE) of the MLP classifier in classifying query from prompt context cells. We use $\lambda_{\mathrm{CLS}}=1$.

#### 4.2.5. Generative procedure

The post-training setup closely resembles a conditional mask diffusion model, where prompt cells represent unmasked tokens and query cell represent masked tokens [mask] that the model must predict. Two key differences distinguish Stack’s generative inference:

1. The gene expression profile of query cells is inputted to the model in order to encode cell type information. Combined with $P_{\text{query}}$, it serves a similar role to positional encodings of [mask] tokens in language models.
2. Masked language models derive token-level confidence directly from softmax probabilities over the vocabulary. Since our output space is continuous, we instead train a separate classifier to estimate prediction confidence, which guides the selective unmasking procedure during generation.

At each generative step, the model receives mini-batches containing concatenated prompt condition cells, prompt context cells and query cells. Here, prompt context cells correspond to original cell expression profiles. The ratio of prompt condition cells remains constant at 25%, while the ratio of prompt context cells increases linearly from 0.2 to 0.4 throughout the generative process. A boolean array is_mask is maintained to indicate whether each query position remains to be predicted, and is initialized with all True for query cells. Each step consists of a prediction-and-update cycle guided by a linear masking schedule (1−t∕T):

- **Prediction:** The model performs a forward pass, generating a complete expression profile for all query cells.
- **Confidence Scoring:** The classifier module assesses each predicted query cell, and outputs a logit vector. Positive values indicate the cell is more like a query cell, negative values indicate the cell is more like a prompt cell.
- **Selective Unmasking:** Based on the masking schedule, a fraction of query cells with is_mask=True are selected to be replaced with model prediction. The cells with the lowest logit values are chosen for the replacement.
- **State Update:** The replaced cells’ is_mask values are set to False. Next, all query cells with logit value > 0 (i.e., those classified as query cells) are (re-)set to is_mask=True.

The iterative process concludes when the masking rate in the schedule reaches zero. The final output from this step constitutes the complete, generated expression matrix for the initial set of query cells. The final logit vector is also a part of the output for quantifying generation quality and interpretation.

### 4.3. Model implementation

The Stack model is implemented in PyTorch and trained using the PyTorch Lightning framework. The training process consists of two stages: self-supervised pre-training and supervised post-training for in-context prompting.

#### 4.3.1. Base model

The Stack architecture consists of $N_{L}\in \{6,9\}$ tabular transformer layers. Each cell is tokenized into *n* = 100 tokens, each with dimension d∈{8,16,32}, yielding a total per-cell embedding dimension nd∈{800,1600,3200}. The input to the transformer is a cell set of K=256 cells tokenized. The feed-forward network within each layer uses a GELU activation function. The decoder is implemented as a 2-layer MLP with GELU activation. No dropout was applied during pre-training. Configurations of different model settings are detailed in the scaling study section.

#### 4.3.2. Pre-training data

The pre-training data was sourced from the full human scBaseCount, the scBaseCount subset, or the human CELLxGENE. For scBaseCount, we filtered cells with 300–7,000 detected genes and at least 700 UMIs. No filtering was performed for CELLxGENE datasets. For model training, a unified gene list was created by computing the union of the top 1,000 highly variable genes (HVGs) from each scBaseCount data file, capped at a maximum of 15,012 total genes. HVGs were identified using analytic Pearson residuals (Lause et al., 2021). The dataloader generates samples by creating non-overlapping, contiguous chunks of *K* = 256 cells from each file. Cells in chunks shorter than *K* are dropped. Pre-training data metrics are summarized in Table 2.

#### 4.3.3. Pre-training setup

The Stack base/large model was pre-trained for 10 epochs using the AdamW optimizer with a peak learning rate of 1 × 10^−4^ and a weight decay of 3 × 10^−3^. For Stack XLarge and Huge models, the peak learning rate was tuned down to 3 × 10^−5^ to stabilize training. A cosine annealing learning rate schedule was used with a linear warmup over the first epoch. The Sliced Wasserstein regularization weight $\lambda_{\mathrm{SW}}$ was set to 0.01. All experiments were conducted on a single NVIDIA H100 GPU (80GB HBM) with 320GB system RAM, using bf16 mixed precision with a batch size of 32 and 4 training data-loading workers. We benchmarked data-loading and pre-training speed against a State Embedding model under equivalent settings.

#### 4.3.4. Post-training data

The dataset for supervised alignment was curated from multiple public sources, including the Parse 10M PBMC data (Parse Biosciences, 2023), and a programmatically selected subset of the CELLxGENE database (Program et al., 2025). To ensure suitability for learning donor-specific effects, a CELLxGENE subset was filtered to include only large-scale datasets (> 50, 000 cells) with at least five unique donors. As the default "cell_type" column in CELLxGENE is found to be suboptimal, we implemented an automatic procedure to identify optimal cell type annotation column for each dataset, employing a heuristic algorithm that prioritizes author-provided, intermediate-granularity labels (e.g., ‘author_cell_type’, ‘ann_coarse’) over standardized ontologies or overly detailed subtypes. The dataset selection procedure resulted in 45 million cells from 189 datasets. The posttraining dataloader first splits the curated datasets into training, validation, and test sets based on donor or sample ID. Each training sample consists of a cell set of *K* = 512 cells, which is further partitioned into $K_{\text{kept}}=128$ prompt condition cells and $K_{\text{query}}=384$ prompt context/target cells.

#### 4.3.5. Post-training setup

The model was fine-tuned for 8 epochs, starting from the pre-trained weights from the Stack (large) model trained on the full human scBaseCount. We used the AdamW optimizer with a peak learning rate of 2 × 10^−5^ and a weight decay of 3 × 10^−3^. A cosine annealing learning rate schedule with a 1-epoch linear warmup and min learning rate 5 × 10^−6^ was applied. For teacher model updates, we employed an exponential moving average (EMA) with a decay rate of 0.95, applied every 500 optimization steps. The training was configured with a batch size of 8 and 4 steps of gradient accumulation, resulting in an effective batch size of 32. Fine-tuning experiments were conducted on a single NVIDIA H100 GPU (80GB HBM) with 400GB system RAM, using bf16 mixed precision.

### 4.4. Ablation and scaling studies

We performed comprehensive ablation and scaling studies to evaluate the performance of Stack across settings and scales. An overview of tested models is shown below. All models use hidden dimension *d* = 100 and attention heads $N_{H_{C}}=N_{H_{G}}=8$, except for XLarge and Huge models which use $N_{H_{C}}=20$.

We also evaluated two ablations of Stack (Base) †: (i) without latent regularization, and (ii) without both latent regularization and inter-cellular attention.

### 4.5. Stack embedding evaluations

#### 4.5.1. Probing evaluation

To assess the biological information encoded in the learned cell representations, we implemented a multitiered probing framework. All probing experiments employed a group-based splitting strategy, where datasets were partitioned by donor ID for observational data and PBMC perturbation data, or by 50% sample split (grouped by library label) for cell-line perturbational data. This ensures that models are evaluated on cells from entirely unseen donors (or library splits for cell-line datasets). To address class imbalance in the test set, we capped the maximum contribution per donor at 2,000 cells for observational data, 25,000 cells for Parse, and 100,000 cells per sample split for other perturbation datasets. Two main probing approaches were employed to evaluate different aspects of the learned representations:

- **Linear probing:** Logistic regression for classification tasks and ridge regression for continuous variables, trained with 5-fold cross-validation on 80% of donors. This setting trains separate models for each cell type, enabling assessment of cell-type-specific information encoding. All analyses were performed on the top 20 most abundant cell types if the total cell type number exceeds 20 (or 5 per tissue in Tabula Sapiens).
- **MLP probe:** For capturing non-linear relationships, we implemented an MLP consisting of an input layer normalization, a hidden layer with 128 units and ReLU activation, dropout of 0.2, and a task-specific output layer. The model was optimized using AdamW with learning rate 1 × 10^−3^ for up to 80 epochs with early stopping (patience of 12 epochs). L2 regularization strength was selected via grid search over 0, 10^−5^, 10^−4^, 10^−3^, 10^−2^, 10^−1^} based on validation loss, using a 70/15/15 train/validation/test split by donor. The model is trained on 5 most abundant cell types together for each dataset.

Performance was evaluated at the single-cell level. Since the test set caps each donor’s contribution, the calculated cell-level accuracy metrics equal donor-level average metrics when all donors exceed the 2,000-cell threshold, and smoothly approximate it when some donors have fewer cells. For classification tasks, we report balanced accuracy. For regression tasks, we report Pearson correlation coefficients. All evaluations were performed with a fixed random seed of 42.

#### 4.5.2. Batch integration evaluation

We performed batch integration evaluations using the scib-metrics package (v0.5.5). To remove extremely rare cell populations that lead to benchmarking errors, the analysis was conducted on a subset containing the top 20 most abundant cell types, or the top 5 most abundant cell classes for Tabula Sapiens. If the resulting cell count exceeded 100,000, the subset was randomly downsampled to this size. The evaluation was structured around two primary objectives, each assessed by a specific suite of metrics.

1. **Batch Correction:** To quantify the removal of technical batch effects, we measured the k-nearest neighbor Batch Effect Test (kBET), graph connectivity, principal component regression (PCR) score, integration Local Inverse Simpson’s Index (iLISI), and batch-removal-adapted silhouette (BRAS) (Rautenstrauch and Ohler, 2025).
2. **Biological Conservation:** To assess preservation of biological signal, we measured Normalized Mutual Information (NMI) and Adjusted Rand Index (ARI) of Leiden clusters against ground-truth cell type labels, silhouette score for cell type labels, and the cell type Local Inverse Simpson’s Index (cLISI).

These scores are aggregated to compute the total score, following the scib-metrics default (Luecken et al., 2022).

#### 4.5.3. Baseline models

The Stack embedding was benchmarked against several methods:

1. **PC HVG:** We performed library normalization, log1p transformation, and Scanpy default highly variable gene selection to identify the top 2,000 highly variable genes. These genes were then reduced to 800/50 principal components. The former setting, matching the dimensionality of Stack (base), excels at probing tasks, while the latter is better for integration evaluations.
2. **scGPT (v0.2.4):** We used the recommended whole-human scGPT checkpoint to generate cell embeddings. Input data was preprocessed with library size normalization and log1p transformation, with a batch size of 256 for inference.
3. **UCE (commit 8227a65):** We employed the 33-layer model checkpoint in (Rosen et al., 2023) with batch size 20 to extract embeddings. The model operates directly on raw count data without normalization. As UCE by default can skip an extremely small amount of cells due to preprocessing, we aligned the output embeddings back to the original cell indices, filling missing cells with zeros.
4. **State (SE) (state v0.9.27):** We used the SE-600M checkpoint on huggingface and the state emb transform command to infer embeddings on raw count data. Data loader throughput was measured using: uv run state emb fit model.batch_size=32 optimizer.gradient_accumulation_steps=256 dataset.num_train_workers=4.
5. **TranscriptFormer (v0.6.1):** We used the TF-sapiens checkpoint from (Pearce et al., 2025) due to its advantage on human scRNA-seq data evaluations. We used the provided CLI to infer embeddings from raw count data.
6. **scVI fine-tuned (scvi-tools v1.3.1):** Pre-trained scVI model initially trained on the scBaseCount subset for 10 epochs, with 2 hidden layers of 2000 hidden dimensions and 800 latent dimensions, or 256 hidden dimensions and 50 latent dimensions. The model was subsequently fine-tuned on each target dataset for 20 additional epochs with learning rate 5 × 10^−4^. The fine-tuning employed a streaming mini-batch strategy: samples of 256 cells were drawn from individual files and concatenated to form training batches of 4096 cells. Each cell was tagged with a batch ID derived from its source file. The strategy greatly accelerates scVI training on large single-cell data collections.
7. **scVI from scratch (scvi-tools v1.3.1):** Models were configured with 2 hidden layers of 2000/128 hidden dimensions and 800/30 latent dimensions, and trained for 50 epochs using Adam optimizer with learning rate 1 × 10^−3^ and weight decay 1 × 10^−4^.

#### 4.5.4. Evaluation datasets

The five observational studies for probing and integration evaluations are downloaded from the CELLxGENE portal (Program et al., 2025) (Kidney, BCL, SEAS-AD MTG, LUCA, and Tabula Sapiens). Perturbation datasets are downloaded from their official websites (Tahoe, Parse, X-Atlas:Orion (Xaira), and the OpenProblems competition). We balanced the tissue composition of Tabula Sapiens, downsampling cell in each tissue to 20,000 if the cell number exceeds the threshold.

### 4.6. Cell prompting task evaluation

The prompting evaluation framework assesses how well a model can generate desired expression profiles given prompt and query cells through in-context learning (ICL). We evaluated four ICL settings:

#### 4.6.1. Evaluation data construction

In settings with cell type hold-outs (1, 3, 4), a set of broad cell classes present in one donor/condition was randomly sampled and held out (ratio 0.75) as target cells to be predicted. The remaining non-held-out cell types from this donor formed the prompt data, while held-out cell types from another donor/condition were used as query cells. In response prediction across samples (settings 2), all data were subsetted to a single cell type (T cells). In perturbational data (settings 1–2), control conditions from the same prompt donor are typically available. Therefore, we additionally utilized these cells as auxiliary prompts to the model and used the output as a “synthetic control” for cell-eval benchmarking. This synthetic control approach was applied to Stack and other baselines when appropriate. For observational prompting tasks, oracle baselines additionally used non-held-out cell types in query conditions as auxiliary data, which were not available to Stack. We equalized the number of query, auxiliary, and target cells by downsampling to the minimum available cell count among all three groups. To account for rare cell types in settings 1 and 3, we upsampled each cell type in the query data to a minimum of 2000 and 1000 cells, respectively.

#### 4.6.2. Baseline models

The prompting performance of Stack was benchmarked against several methods:

1. **Original Query:** The original query cells are used for prediction, representing a zero-change scenario where no information from the prompt is used.
2. **Nearest Cell Type in Prompt:** This baseline computes pseudobulk expression profiles for all cell types in the prompt data. For each query cell, it identifies the most similar cell type in the prompt based on pseudobulk Pearson correlation, then assigns an expression profile from a randomly sampled cell of that matched cell type without replacement. Once all cells from a cell type are exhausted, the second-closest cell type is used, and so on.
3. **Same Cell Type in Prompt:** This baseline assigns expression profiles by sampling from cells of the same cell type in the prompt without replacement when possible.
4. **PerturbMean/DonorMean:** This method computes the average difference in expression profiles (per cell type) between the prompt and query contexts. This difference vector is calculated on non-held-out cell types between prompt and auxiliary data, then added to the expression profiles of the query cells. The synthetic control is not applicable for this baseline, as it would be equivalent to the query data.
5. **scVI:** A 2-layer scVI model (scvi-tools v1.3.1) with 30 latent dimensions and 200 hidden dimensions was trained on combined prompt and auxiliary samples, using dataset origin as the batch key, for 50 epochs. The model was then used to project query cells into the latent space and sample their expression profiles, artificially specifying “prompt” as the batch label.
6. **State:** State models (v0.9.27) were trained using the ST+SE setting (Adduri et al., 2025), where the model predicts cell embeddings and simultaneously decodes them back to gene expression space. Models were trained with Maximum Mean Discrepancy (MMD) loss on prompt, query, and auxiliary data, using the union of highly variable genes in cell-eval benchmarks across random seeds. Only control cells from the query set were used for model training. All models used a hidden dimension of 328 and cell set length of 32. Models were for 60,000 steps (batch size 8, learning rate 3 × 10^−4^). Random basal mapping was employed with gene-space outputs. The best checkpoints were selected via validation loss on held out cells.

#### 4.6.3. Evaluation metrics

We evaluated the generated expression profiles against ground truth data using pseudobulk correlation and DE metrics. Both categories of metrics are implemented using cell-eval v0.6.6 (Adduri et al., 2025) with default parameters, employing Wilcoxon rank-sum tests for DE detection and Benjamini-Hochberg correction for multiple testing. All metrics were computed on top 2000 log-normalized highly variable genes, identified from the concatenation of target and query data. For setting 4, we additionally evaluated the integration performance of ground truth and predicted gene expression profiles using scIB, with the same configuration as the earlier benchmarking.

- **Pseudobulk correlation.** We measured how well methods capture pseudo-bulk level perturbation effects by two metrics:
  - **Pearson Delta**: Pearson correlation between predicted and observed expression changes. For each perturbation t, we calculate the expression delta as $\Delta_{t}=|p_{t}-p_{\mathrm{ctrl}}|$ for both predicted (${\bar{\Delta }}_{t}$) and ground truth ($\Delta_{t}$) pseudobulks, then compute: Pearson-$\Delta =\mathrm{corr}({\bar{\Delta }}_{t},\Delta_{t})$
  - **DE Spearman LFC**: the Spearman rank correlation between predicted and observed log fold changes, calculated within the set of significantly differentially expressed (DE) genes in the ground truth.
  - **DE direction match**: This metric measures whether the predicted direction of gene expression change (i.e., up- or down-regulation) matches the ground truth. It is calculated only on the set of genes that are significantly differentially expressed (DE) in both the predicted and true data. The score is the fraction of these shared DE genes for which the direction of change matches. This metric replaces Pearson Delta in observational prompting tasks. Pearson Delta performs comparably to DE Spearman LFC when many genes are identified as DEGs, while also showing greater vulnerability to batch effects.
- **Differential expression accuracy.** Finally, we evaluated whether the prediction captures differential expression between ground truth data and the input query condition.
  - **PR-AUC**: Area under precision-recall curve using binary DE labels and $-\log_{10}(p\text{-values})$ as scores, using sklearn average precision score implementation.
  - **Spearman effect size**: To compare the relative effect sizes of perturbations, we calculate Spearman correlation coefficients on the number of differentially expressed genes (adjusted p-value < 0.05) between predicted and ground truth. This assesses whether models accurately capture relative effect sizes across different conditions. We averaged predictions across random seeds within each cell type and perturbation/donor, yielding a Spearman correlation computed across perturbations/donors per cell type.
  - **DE Overlap Accuracy**: Computes the overlap between the top-*N* genes from the true DE abs-log-fold-change ranking and the top-*N* genes from the predicted DE ranking, calculated as ∣top-*N* true∩ top-*N* predicted∣/*N*, where *N* is the total number of true DE genes.
  - **DE Precision-at-***N*: Computes the overlap between the top-*N* genes from the true DE ranking and the top-*N* genes from the predicted DE ranking, calculated as ∣top-*N* true ∩ top-*N* predicted∣/*N*, where *N* is the total number of predicted DE genes. This metric assesses the fidelity of predicted DE gene lists and replaces Spearman effect size correlation in Setting 2, where the limited number of perturbations with similar effect sizes makes rank-based correlation unreliable.
  - **Jaccard similarity**: For each perturbation, we compute the Jaccard index between the set of predicted DE genes and ground truth DE genes, defined as the size of the intersection divided by the size of the union of the two sets.

#### 4.6.4. Evaluation datasets

For evaluations, we used 1. OpenProblems drug perturbation (Luecken et al., 2025), 2. Cytokine stimulation (Dong et al., 2023), 3. Immune aging (Wells et al., 2025), 4. Tabula Sapiens (Consortium* et al., 2022), 5. Kidney atlas (De Boer et al., 2021), 6. Lymph node BCL (Li et al., 2025), 7. Liver atlas (Edgar et al., 2025), 8. Parse cytokine perturbation dataset (Parse Biosciences, 2023). Observational atlases (3-7) were downloaded from the CELLxGENE portal, and the cytokine stimulation data was downloaded from Dryad (Dong et al., 2023). The cytokine stimulation data (Dong et al., 2023) comprise 3 donors, but donor 1 has very limited cell numbers. Therefore, we subsetted the dataset to include only donors 2 and 3 (relabeled as donors a and b) and only included cells from the acute condition (2 days).

For setting 1, the OpenProblems dataset used prompts and queries sampled from the same donor (3 donors total) within each test experiment. For settings 2, prompts used T cells from one Parse donor under cytokine conditions (IFN-*β*, IFN-*γ*, IL-6, TNF-*α*), while queries used control T cells from donors a or b in (Dong et al., 2023). Ground truth evaluation used corresponding individual and combination conditions (IFN-*β*, IFN-*γ*, IL-6, TNF-*α*, IFN-*β*+IFN-*γ*, IFN-*β*+IL-6, IFN-*β*+TNF-*α*) from (Dong et al., 2023). For combinatorial perturbation evaluations, we computed weighted sums of single-perturbation Stack predictions. Due to dosage differences between the Parse dataset and Dong et al. (2023), we performed a grid search over weights [0.1, 0.3, 0.5, 0.7, 0.9] to determine the optimal weighted average of normalized gene expression conditions in the original prompt T cells. The optimal weight was selected based on Pearson Delta and subsequently used to generate Stack predictions for combinatorial perturbations. For setting 3, prompts and queries comprised non-overlapping cell types sampled from different donors in each dataset. For setting 4, prompts comprised: Parse control cells (PBS condition) with one donor sampled per prompt; Parse donor 1 cells with one perturbation sampled per prompt; immune aging dataset cells with one donor per prompt; OpenProblems cells with one perturbation per prompt. All queries in this setting used immune cells from Tabula Sapiens. In settings 3 and 4, we performed cell class-level hold-out for each evaluated dataset, relabeling cell annotations from original author annotations to align with the broad cell classes defined in Tabula Sapiens for corresponding tissues. Holding out at the cell class level minimizes potential information leakage across similar cell types.

### 4.7. Whole-organism perturbational atlas *Perturb Sapiens*

In the analysis, we used each condition from donor 2 in OpenProblems drug perturbation data and donor 1 in Parse cytokine perturbation data as the prompt, and the tissue balanced version of Tabula Sapiens as the query. We used post-trained Stack (T=5) to perform in-context generation. We removed cells with classifier logits above 2.5.

We generated log fold change heatmaps for individual cytokine treatments. For each cell type, up to 10,000 cells per condition were subsampled, normalized, and log-transformed. Genes were subsetted to 4,000 highly variable genes identified via Pearson residual in the example ADSF *Perturb Sapiens* dataset (Lause et al., 2021). Differential expression analysis was performed using the Wilcoxon rank-sum test with Benjamini-Hochberg correction (DEG: FDR < 0.05, ∣log_2_FC∣ > 0.25/0.5 for Parse/OpenProblems respectively). To compare *Perturb Sapiens* performance against biological replicates across drug or cytokine perturbations, the same differential expression procedure was applied, with each condition subsampled to a maximum of 15,000 cells. Cell-eval evaluation metrics were constructed as described above, with one modification: significant genes were defined using an explicit minimum log fold change threshold, consistent with the DEG procedure.

For evaluation on non-immune cells, we downloaded four epithelial in vitro cytokine perturbation datasets from the GEO database (Koh et al., 2023; Swindell et al., 2018; Lee et al., 2022; Saito et al., 2021). All evaluations were restricted to the top 4,000 highly variable genes identified from the example ADSF *Perturb Sapiens*. For the first three datasets, log fold changes, *p*-values, and adjusted *p*-values were computed using PyDESeq2 (v0.5.2) on either pseudobulk (Koh et al., 2023) or bulk RNA-seq data (Swindell et al., 2018; Lee et al., 2022). For (Saito et al., 2021), due to the normalized TPM format of the deposited data, we performed a donor-wise paired *t*-test to calculate log fold changes, *p*-values, and adjusted *p*-values using Benjamini–Hochberg correction. Differential expression analysis on *Perturb Sapiens* was performed using Scanpy’s Wilcoxon rank-sum test implementation. Airway and keratinocyte epithelial populations in *Perturb Sapiens* were constructed by selecting cell classes annotated as “epithelial” from their respective tissues (lung, trachea for airway; skin and tongue for keratinocytes). To ensure fair comparison across *Perturb Sapiens* cell types, each cell type population was capped at a maximum of 15,000 cells. We applied an LFC threshold of 0.5 for *in vitro* single-cell data evaluations and 0.25 for bulk data evaluations. Finally, for each drug/cytokine perturbation and its matched control, we identified tissue–cell type combinations with sufficient cell counts (≥ 1000 cells). We then applied the same differential expression analysis pipeline described above to compute log-fold changes and adjusted *p*-values. In Figs. S17 and S18, perturbation effects are defined as log fold changes, with non-significant values (adjusted p > 0.05) set to zero.

## Supplementary Material

Supplement 1

## Figures and Tables

**Table 1 ∣:** Components of the post-training input cell set $X_{\text{in}}$.

Component	Notation	Size	Description
Prompt condition	$X_{\text{prompt}}^{\text{fixed}}$	0.25K	Original cells from prompt sample
Prompt context	${\hat{X}}_{\text{prompt}}^{\text{fixed}}$	$K_{\text{kept}}$	Sampled from teacher-predicted distributions
Query	$X_{\text{query}}$	$K_{\text{query}}$	Cells from a different biological context

*Note:* $K_{\text{kept}}+K_{\text{query}}=0.75K$; ratio $K_{\text{kept}}∕(K_{\text{kept}}+K_{\text{query}})∼𝒰(0,1)$.

**Table 2 ∣:** An overview of training datasets for Stack models in this study.

Training sets	#Training Datasets	#Training Cells
CellXGene (§)	905	73.7M
scBaseCount subset (†)	9004	60.2M
scBaseCount full set (‡)	19978	148.8M

**Table 3 ∣:** An overview of model settings tested in the scaling study.

Model Variant	Layers $N_{L}$	Total Embed Dim nd	Params	Non-emb Params
Stack (Base−) †	3	800	69.1M	57.0M
Stack (Base) §, †, ‡	6	800	76.7M	64.7M
Stack (Base+) †	9	800	84.4M	72.4M
Stack (Medium) †	6	1600	186M	163M
Stack (Large) †, ‡	9	1600	217M	193M
Stack (XLarge) ‡	6	3200	506M	459M
Stack (Huge) ‡	9	3200	629M	582M

**Table 4 ∣:** Overview of datasets used in this study. * indicates subsampling from the original dataset.

Dataset Category	Dataset Name	# Donors	# Cell Types	Conditions
Observational Atlases	Kidney atlas	77	43	Disease: 14 (AKI) / 37 (CKD) / 26 (Healthy) Hypertension: 41 (Yes) / 36 (No) Diabetes history: 38 (Yes) / 38 (No)
	Brain atlas (SEAS MTG)	38	65	Microinfarct pathology: 34 (0–3) / 2 (4–6) / 2 (7–10) ADNC: 4 (Not AD) / 7 (Low) / 9 (Intermediate) / 18 (High) Braak stage: 2 (0) / 2 (II) / 4 (III) / 8 (IV) / 11 (V) / 11 (VI) Thal phase: 4 (0) / 3 (1) /4 (2) / 7 (3) / 10 (4) / 10 (5) CERAD score: 9 (Absent) / 5 (Sparse) / 7 (Moderate) / 17 (Frequent) APOE4 status: 23 (N) / 15 (Y)
	Lymph node atlas (BCL)	223	21	Disease: 208 (BCL) / 15 (Healthy) LymphoMAP: 84 (FMAC) / 76 (LN) / 64 (TEX)
	Lung atlas (LUCA)	160	39	Disease: 18 (COPD) / 75 (LUAD) / 17 (LUSC) / 2 (NSCLC) / 48 (Healthy) UICC stage: 45 (I) / 17 (II) / 7 (III) / 24 (IV) / 66 (non-cancer) Ever smoker: 85 (Yes) / 75 (No)
	Tabula Sapiens	24	31	25 tissues with >1 donors
Perturbational Datasets	OpenProblems-PBMC	3	6	147 conditions
	Tahoe-100M	–	40*	250* perturbations from plates 1-3
	Parse-PBMC	12	17	90 perturbations
	X-Atlas:Orion (Xaira)	–	2	Top 50* perturbations per cell line

**Table 5 ∣:** Overview of in-context cell prompting tasks for Stack.

Task Category	Task Name	Prompt	Query	Same Dataset?
Perturbational ICL	1. Perturbation effect prediction for novel cell types	Perturbed cells (random types)	Control cells (non-overlapping types)	Yes
	2. Perturbation effect prediction for novel samples	Perturbed T cells (Donor A)	Unperturbed T cells (Donor B)	No
Observational ICL	3. Hold-out cell type prediction	Selected cell types (Donor A)	Non-overlapping types (Donor B)	Yes
Hybrid ICL	4. Cross-dataset cell type generation	Selected cell types (Donor/Pert A)	Non-overlapping types (Donor B)	No

## Data Availability

Documentation on accessing scBaseCount can be found at https://github.com/ArcInstitute/arc-virtual-cell-atlas. The generated *Perturb Sapiens* data is deposited on Huggingface: https://huggingface.co/datasets/arcinstitute/Perturb-Sapiens. The CELLxGENE 45M data used for alignment is also available on Huggingface: https://huggingface.co/arcinstitute/Stack-CellxGene45M. The Parse 10M PBMC data is available at the official website https://www.parsebiosciences.com/datasets/10-million-human-pbmcs-in-a-single-experiment/#download. All evaluation data used for this project are publicly available; see the Methods section for download details of other datasets.

## Box

**Box 1.** In-context learning (ICL) tasks for cell prompting.

• **Perturbational ICL** (prompts are perturbed cells and queries are control cells)
1. **Perturbation effect prediction in novel cell types from same sample**: We use randomly sampled perturbed cell types as the prompt, and control cells from remaining cell types as the query. Stack simulates the prompt specified perturbation condition in the query cell types (Fig. 3C-E).
*Example:* Given a PBMC sample where only T cells were perturbed with IL-6, we use perturbed T cells as the prompt and unperturbed B cells/monocytes as the query to predict IL-6 effects on B cells/monocytes.
2. **Perturbation effect prediction in novel samples**: We use one single perturbed cell type (T cells here) as the prompt, and control T cells from another donor in a different dataset as the query. The setting assesses the model’s ability to predict responses observed in the query sample (Fig. 3F).
*Example:* We collected a new PBMC sample and want to predict how T cells respond to IFN-*β* stimulation. We use IFN-*β*-perturbed T cells from a published reference dataset as the prompt, and control T cells from our new sample as the query.
• **Observational ICL** (prompts and queries are cells from observational scRNA-seq samples)
3. **Hold-out cell type prediction**: We use sampled cell types from one donor as the prompt and non-overlapping cell types from another donor in the same dataset as the query. Stack predicts the expression profile of query cell types in the prompt donor (Fig. 3G). This setting evaluates the model’s ability to capture donor-specific expression differences in observational datasets.
*Example:* In a patient cohort, Patient A has profiled macrophages and T cells but lacks fibroblasts due to tissue availability. We use Patient A’s cells as the prompt and fibroblasts from Patient B as the query, to impute Patient A-specific fibroblast expression.
• **Hybrid ICL** (prompts and queries are from different observational or perturbation studies)
4. **Cross-dataset cell type generation**: We use sampled cell types from one condition as the prompt and non-overlapping cell types from another dataset as the query, and predict the query cell type profile in the prompt sample context (Fig. 3H). This setting assesses the model’s capacity to generate cell types absent from an observational atlas or perturbation data of interest.
*Example*: Using drug perturbed T cells and B cells as the prompt, and dendritic cells from an independent scRNA-seq atlas as the query, we predict perturbation responses in dendritic cells that were never experimentally perturbed.

## Code and model availability.

Code for the Stack model is available at https://github.com/ArcInstitute/stack. Model parameters are available on Huggingface: https://huggingface.co/arcinstitute/Stack-Large, https://huggingface.co/arcinstitute/Stack-Large-Aligned.
