# Supplementary material for: Stack: In-Context Learning of Single-Cell Biology
Source: bioRxiv. 2026 Jan 9:2026.01.09.698608. Preprint. [Version 1] doi: 10.64898/2026.01.09.698608 (PMC12803207; doi:10.64898/2026.01.09.698608)
Supplement: Supplement 1 [file NIHPP2026.01.09.698608v1-supplement-1.pdf]

bioRxiv preprint doi: https://doi.org/10.64898/2026.01.09.698608; this version posted January 9, 2026. The copyright holder for this preprint (which was not certified by peer review) is the author/funder, who has granted bioRxiv a license to display the preprint in perpetuity. It is made available under aCC-BY 4.0 International license.

Supplementary Figures and Tables

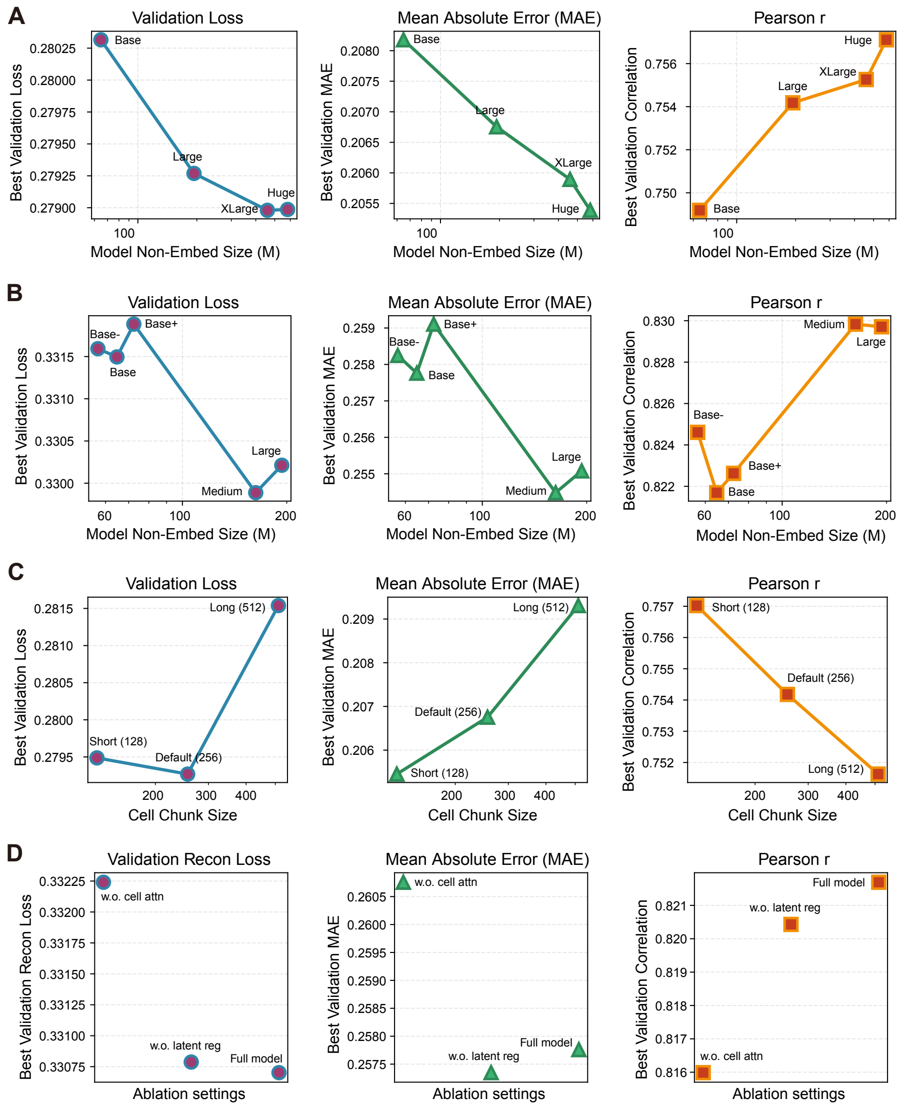

Figure S1 | Scaling and ablation analysis of the Stack model. **A.** Validation performance across model sizes for Stack models trained on the full human scBaseCount (Youngblut et al., 2025). **B.** Validation performance across model sizes for Stack models trained on the scBaseCount subset. **C.** Validation performance across cell set sizes for the Stack (Large) model trained on the full scBaseCount dataset. **D.** Validation performance across ablation settings for Stack (Base) models trained on scBaseCount subset (w.o. latent reg: removing only latent regularization; w.o. cell attn: removing both latent regularization and inter-cellular attention). All models in **A**, **B** and **D** use a cell set size of 256. For validation loss and mean absolute error (MAE), smaller is better; for Pearson r, higher is better.

bioRxiv preprint doi: https://doi.org/10.64898/2026.01.09.698608; this version posted January 9, 2026. The copyright holder for this preprint (which was not certified by peer review) is the author/funder, who has granted bioRxiv a license to display the preprint in perpetuity. It is made available under aCC-BY 4.0 International license.

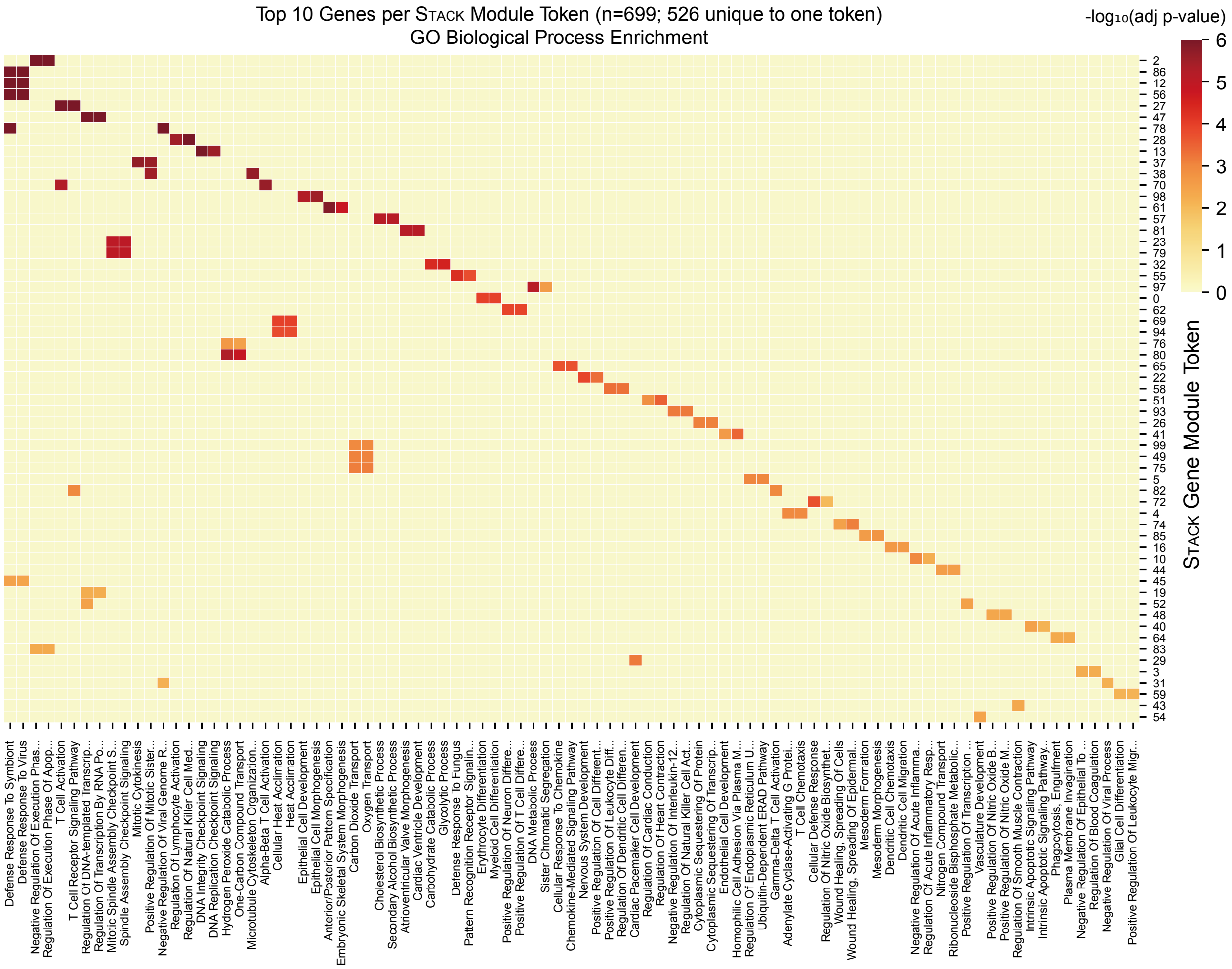

Figure S2 | Adjusted p-value heatmap of Gene Ontology (GO) biological process enrichment analysis for the top 10 most important genes within each STACK (Large) token after pre-training on full human scBaseCount. Gene importance scores were computed by first reshaping the tokenization weight matrix $\mathbf{W}$ to $\mathbb{R}^{n_{\text{hidden}} \times d_{\text{token}} \times n_{\text{genes}}}$, then calculating the mean absolute weight across the token dimension for each hidden module. The top 10 genes per module were selected based on these importance scores. The top 2 enriched pathways per module are shown. Rows (modules) and columns (pathways) were ordered by hierarchical clustering using average linkage with Euclidean distance.

bioRxiv preprint doi: https://doi.org/10.64898/2026.01.09.698608; this version posted January 9, 2026. The copyright holder for this preprint (which was not certified by peer review) is the author/funder, who has granted bioRxiv a license to display the preprint in perpetuity. It is made available under aCC-BY 4.0 International license.

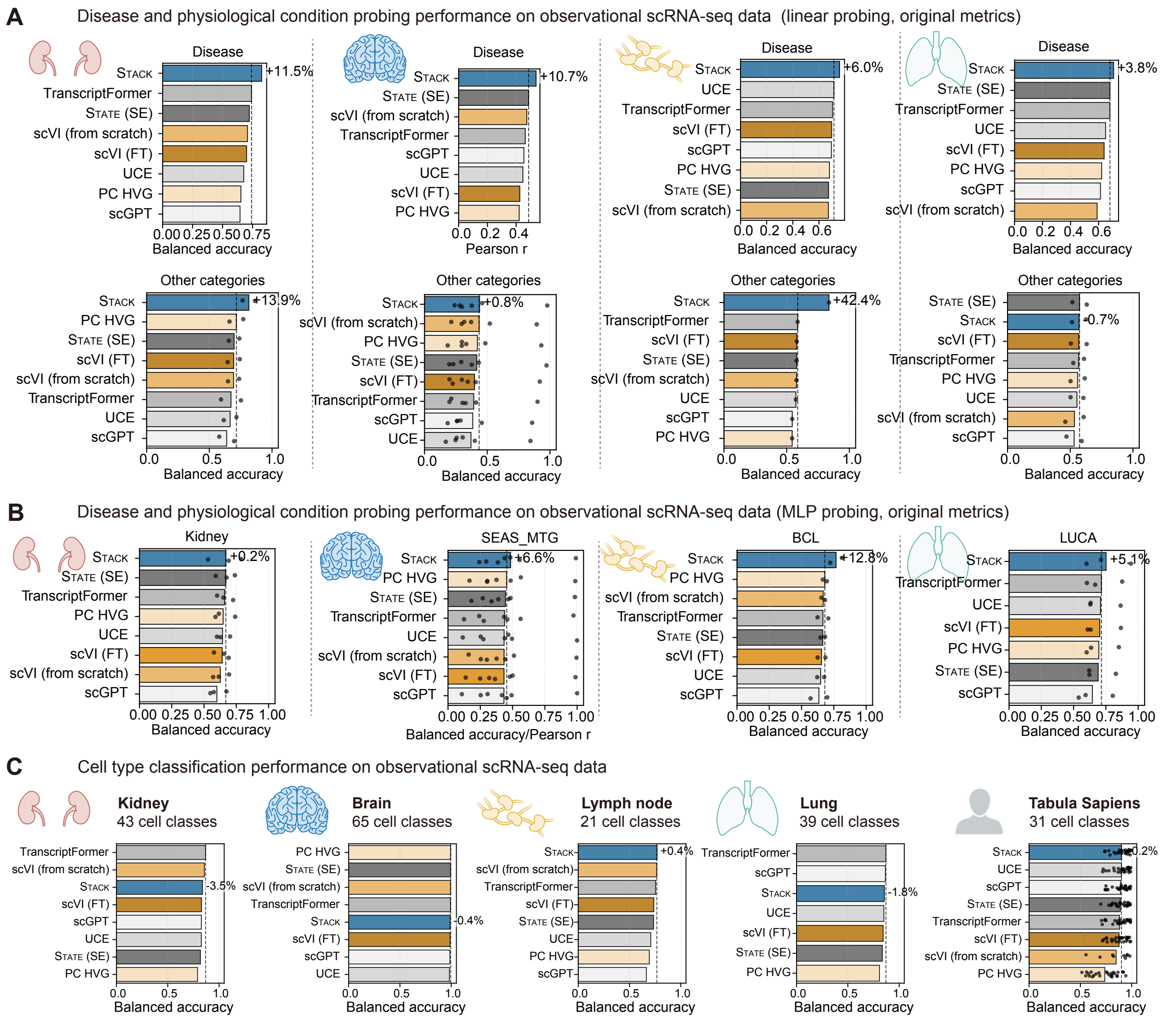

Figure S3 | Additional probing evaluation results. **A.** Disease and physiological condition probing performance on observational scRNA-seq data (De Boer et al., 2021; Li et al., 2025; Salcher et al., 2022; Gabitto et al., 2024). One linear classifier is trained per cell type. The number of experiments per dataset: n=12, 20, 10, 20. See Fig. S4 for full results. All STACK results presented here are based on one model with the (Large) setting pretrained on full human scBaseCount. All panels show (average) values of original metrics (balanced accuracy/Pearson r) without normalization. **B.** Disease and physiological condition MLP probing performance on observational scRNA-seq data. One MLP classifier is trained simultaneously on the top 5 most abundant cell types. See Methods for dataset statistics. **C.** Cell type classification performance on observational scRNA-seq data. In Tabula Sapiens evaluation, each point represents a tissue (n=26).

bioRxiv preprint doi: https://doi.org/10.64898/2026.01.09.698608; this version posted January 9, 2026. The copyright holder for this preprint (which was not certified by peer review) is the author/funder, who has granted bioRxiv a license to display the preprint in perpetuity. It is made available under aCC-BY 4.0 International license.

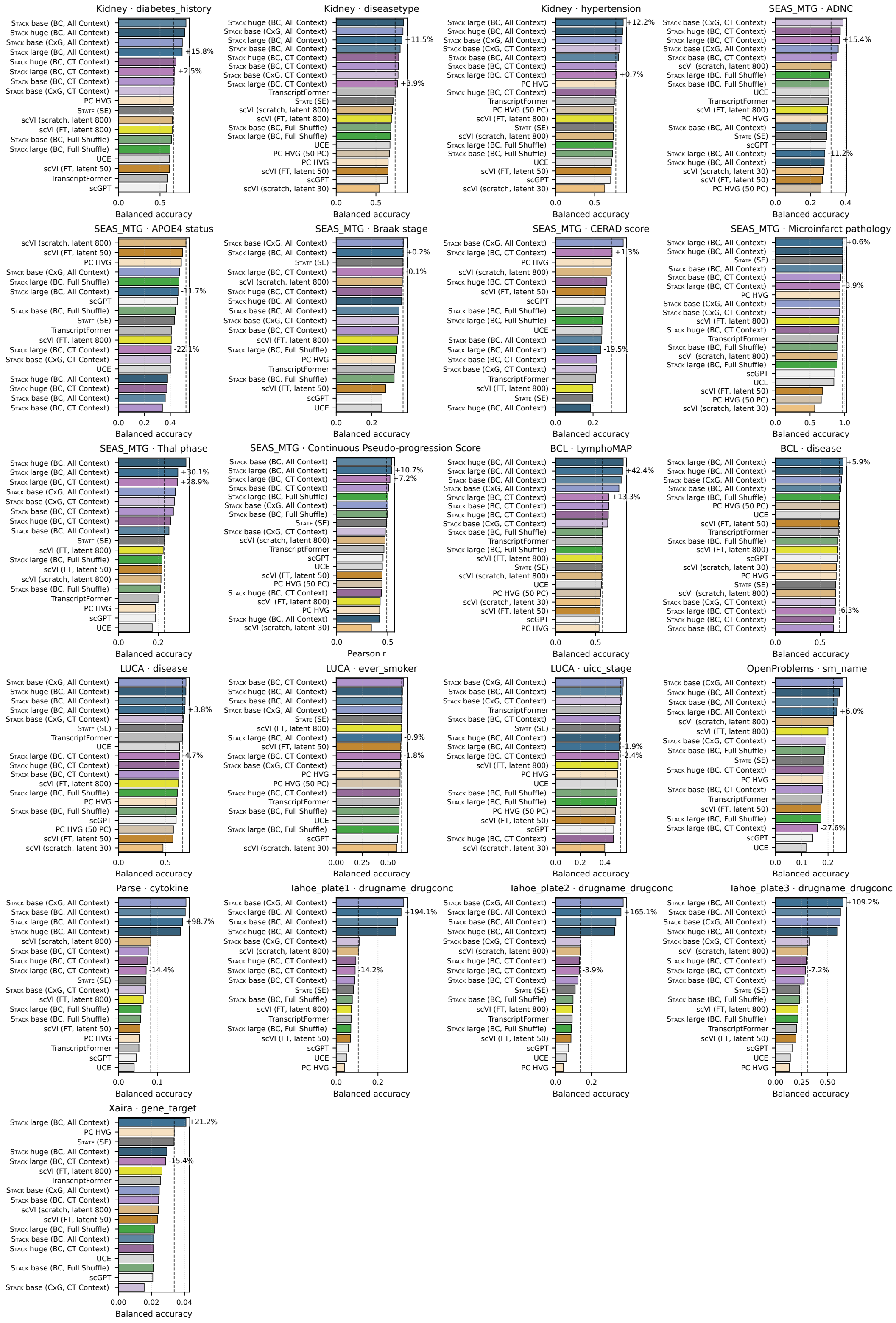

Figure S4 | Per-cell-type linear probing results with additional Stack settings. BC: the model is trained on full human scBaseCount. CxG: the model is trained on CELLxGENE. All Context: Stack uses the default dataset grouping by sample, utilizing all cell types from each sample as context. CT Context: Stack utilizes cells grouped by cell type per sample as context, generates a set of embeddings for each group, then concatenates them to form the total embedding. Full shuffle: The cell order in the evaluation data is randomly shuffled, effectively removing context information. The number of experiments per dataset: n=12, 10, 20, 20, 6, 17, 20, 20, 20, 2.

bioRxiv preprint doi: https://doi.org/10.64898/2026.01.09.698608; this version posted January 9, 2026. The copyright holder for this preprint (which was not certified by peer review) is the author/funder, who has granted bioRxiv a license to display the preprint in perpetuity. It is made available under aCC-BY 4.0 International license.

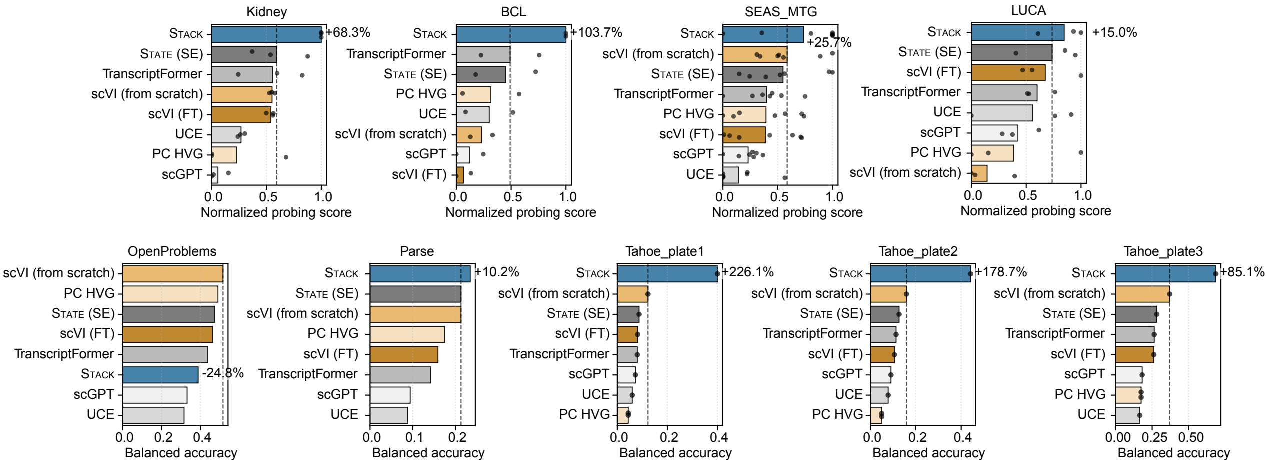

Figure S5 | Linear probing results for each dataset, using the overall best-performing cell type per task. The full results for Xaira are included in Fig. 2.

bioRxiv preprint doi: https://doi.org/10.64898/2026.01.09.698608; this version posted January 9, 2026. The copyright holder for this preprint (which was not certified by peer review) is the author/funder, who has granted bioRxiv a license to display the preprint in perpetuity. It is made available under aCC-BY 4.0 International license.

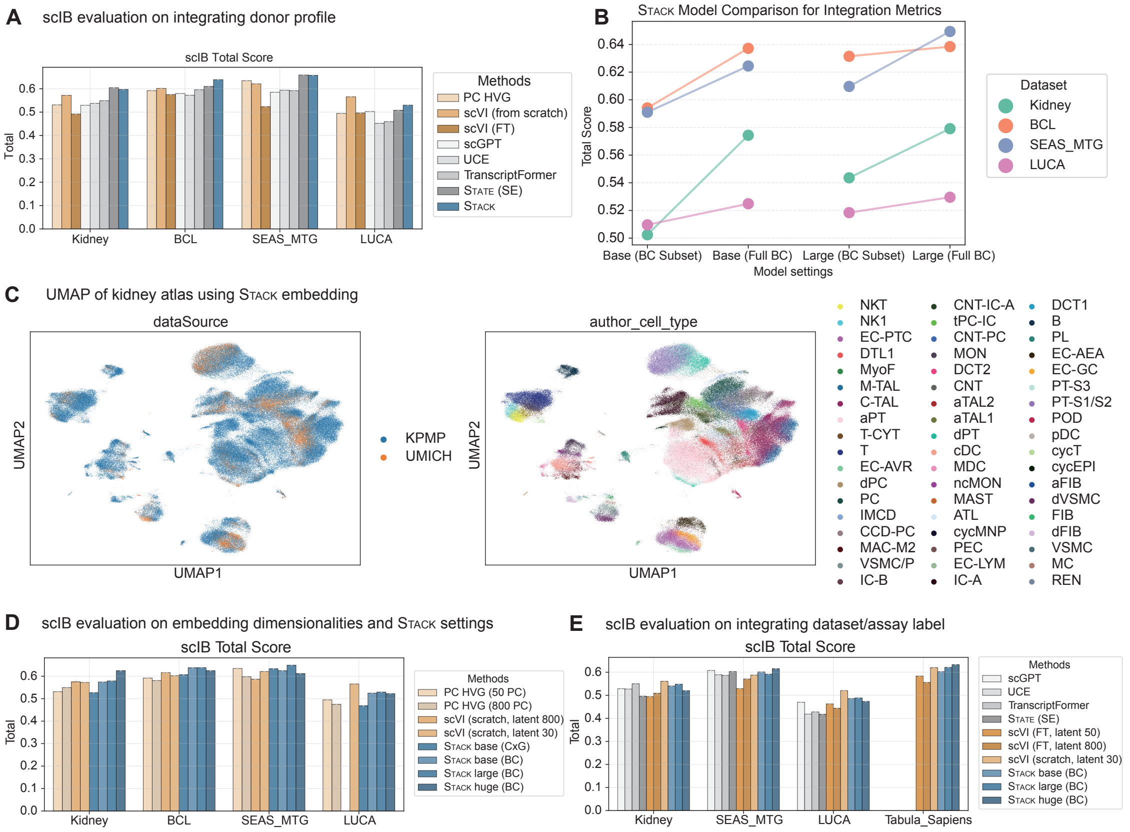

Figure S6 | Additional evaluation results of STACK on batch integration. **A.** scIB evaluation of different methods on integrating donor profiles and preserving cell types (Luecken et al., 2022). Presented STACK results are based on one model with the (Large) setting pretrained on full human scBaseCount.. **B.** scIB batch integration total scores of STACK with different sizes, training data, and evaluation datasets. **C.** UMAP visualization of STACK embedding of the Kidney atlas, colored by data collection and fine-grained cell type. **D.** Comparison of integration performance across different numbers of principal components and scVI latent dimensions. Based on these results, the results of 50 principal components and 30 scVI latent dimensions were selected for main batch integration benchmarks. **E.** Comparison of dataset label integration performance. As BCL involves only a single dataset label, it is not applicable to dataset label integration evaluation. Alternative foundation models (scGPT, UCE, STATE (SE), and TranscriptFormer) were excluded from the Tabula Sapiens evaluation due to their limited performance improvements in the per-tissue benchmark. In **B**, **D**, and **E**, we apply STACK to the full dataset rather than to one sample at a time. This results in a very minor decrease in STACK 's performance. The dataset integration performance in **E** closely matches the donor integration performance shown in **A**.

bioRxiv preprint doi: https://doi.org/10.64898/2026.01.09.698608; this version posted January 9, 2026. The copyright holder for this preprint (which was not certified by peer review) is the author/funder, who has granted bioRxiv a license to display the preprint in perpetuity. It is made available under aCC-BY 4.0 International license.

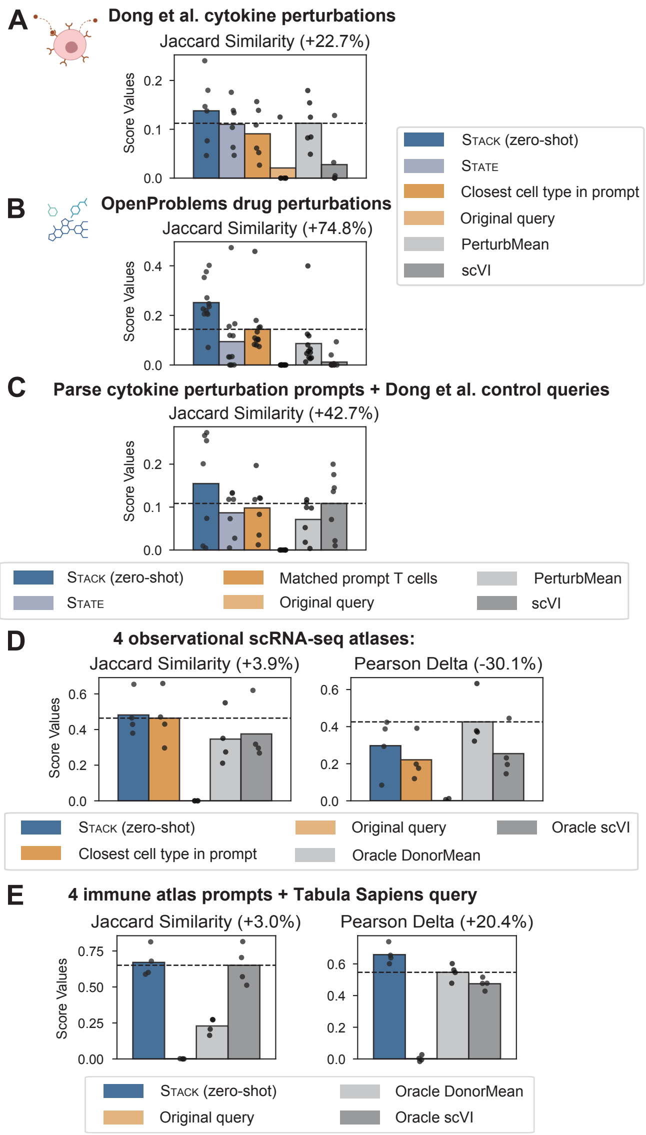

Figure S7 | Additional metrics on cell prompting tasks. **A.** Evaluation of perturbation effect prediction across cell types on the Dong et al. (2023) cytokine perturbation dataset (6 cytokines). **B.** Evaluation of perturbation effect prediction across cell types on the OpenProblems drug perturbation dataset (Luecken et al., 2025) (12 drugs). **C.** Evaluation of T cell response prediction across samples (7 cytokine stimulation conditions). **D.** Evaluation of donor-specific gene expression generation across five atlases. **E.** Evaluation of condition-specific expression generation across four PBMC atlases. See Fig. 3 caption for additional details. For all scores, higher values indicate better performance.

bioRxiv preprint doi: https://doi.org/10.64898/2026.01.09.698608; this version posted January 9, 2026. The copyright holder for this preprint (which was not certified by peer review) is the author/funder, who has granted bioRxiv a license to display the preprint in perpetuity. It is made available under aCC-BY 4.0 International license.

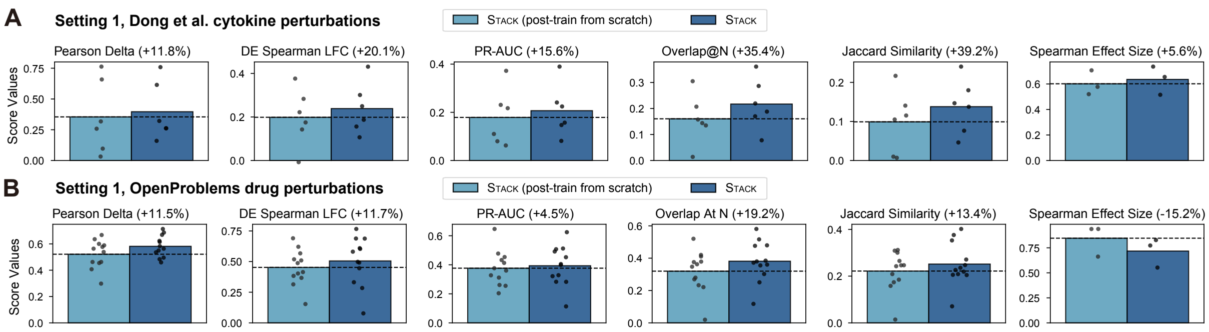

Figure S8 | Comparison of perturbation response prediction in novel cell types between Stack post-trained from pretrained weights and Stack trained from scratch. **A.** Results on the Dong et al. (2023) dataset. **B.** Results on the OpenProblems drug perturbation dataset (Luecken et al., 2025). All percentages shown represent the average performance improvement of Stack over Stack post-trained from scratch.

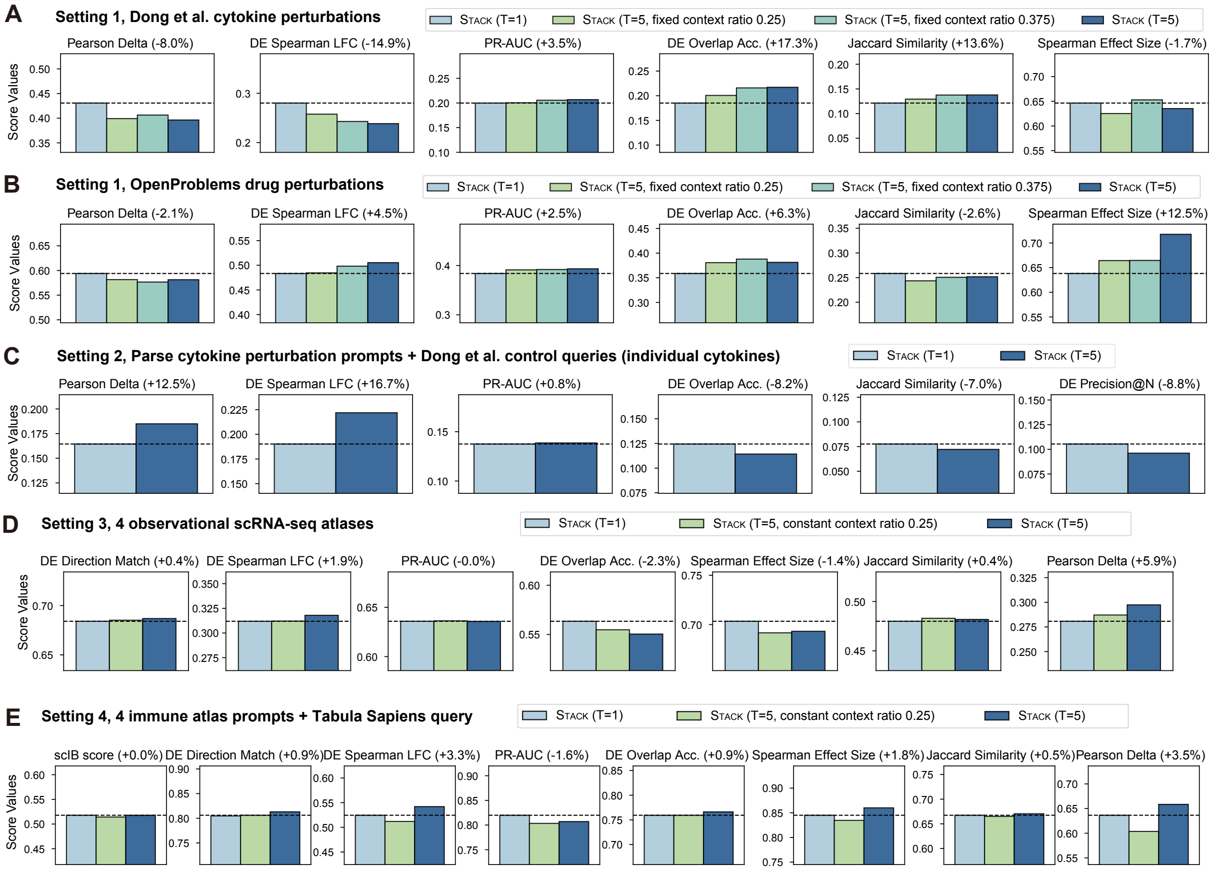

Figure S9 | Evaluations of Stack generative procedure on cell prompting tasks. **A.** Comparison of Stack (T=5) and Stack (T=1) for perturbation effect prediction across cell types on the Dong et al. (2023) dataset. **B.** Comparison of Stack (T=5) and Stack (T=1) for perturbation effect prediction across cell types on the OpenProblems drug perturbation dataset (Luecken et al., 2025). **C.** Evaluation of T cell perturbation response prediction across Parse PBMC and Dong et al. (2023) with individual cytokine stimulation conditions. **D.** Evaluation of donor-specific gene expression generation across five atlases. **E.** Evaluation of condition-specific expression generation across four PBMC atlases as prompts and Tabula Sapiens as queries. All percentages shown represent the average performance improvement of Stack generative settings over predictive settings. See the Fig. 3 caption for the number of experiments and the definition of scores. For all scores, higher values indicate better performance.

bioRxiv preprint doi: https://doi.org/10.64898/2026.01.09.698608; this version posted January 9, 2026. The copyright holder for this preprint (which was not certified by peer review) is the author/funder, who has granted bioRxiv a license to display the preprint in perpetuity. It is made available under aCC-BY 4.0 International license.

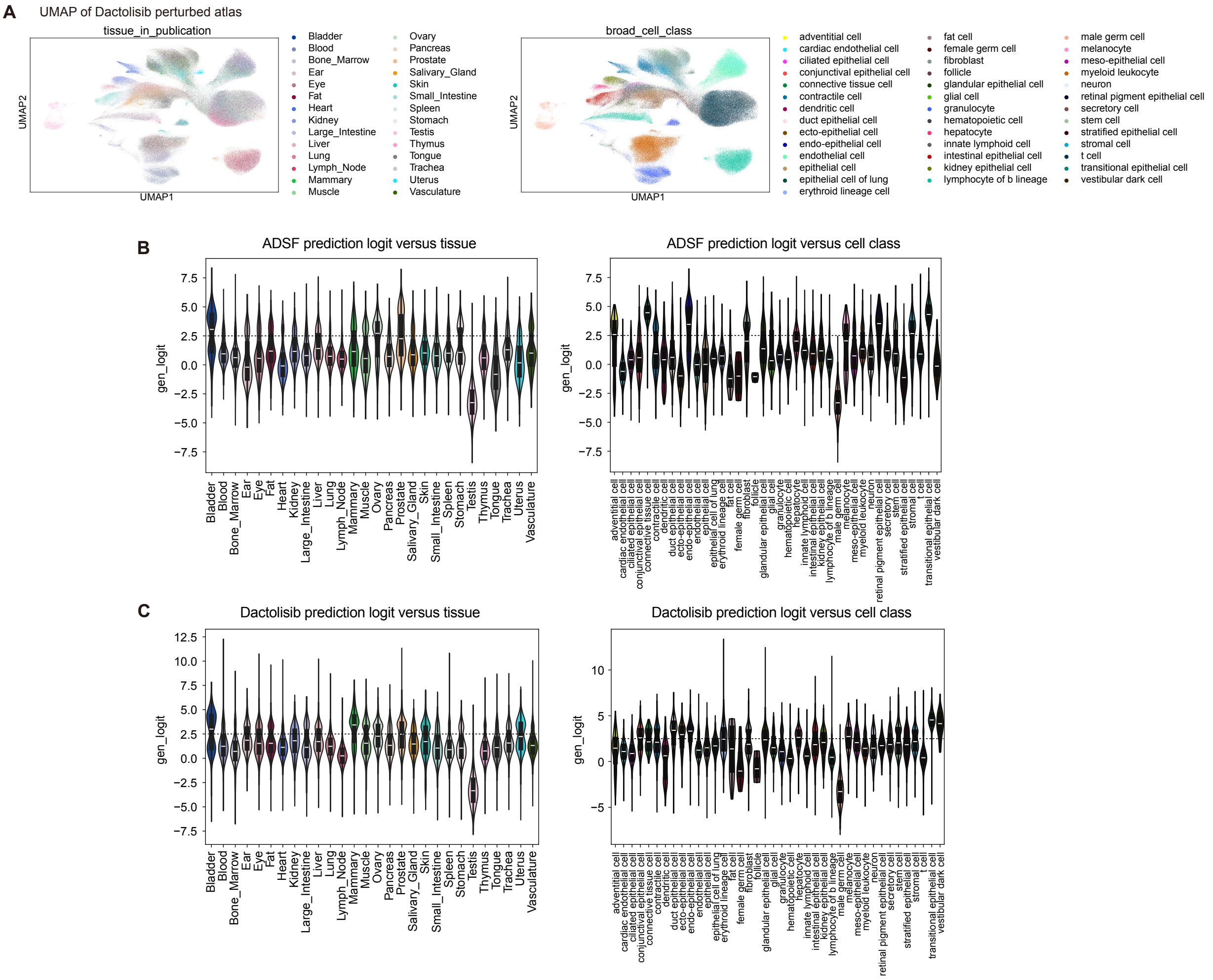

Figure S10 | Additional analysis on *Perturb Sapiens*. **A.** UMAP visualization of STACK embedding of Dactolisib *Perturb Sapiens*, colored by tissue and cell class label. **B.** Violin plots of classifier predicted logit value in ADSF *Perturb Sapiens*, grouped by tissue and cell class. **C.** Violin plots of classifier predicted logit value in Dactolisib *Perturb Sapiens*, grouped by tissue and cell class. Lower logit indicates higher generation confidence.

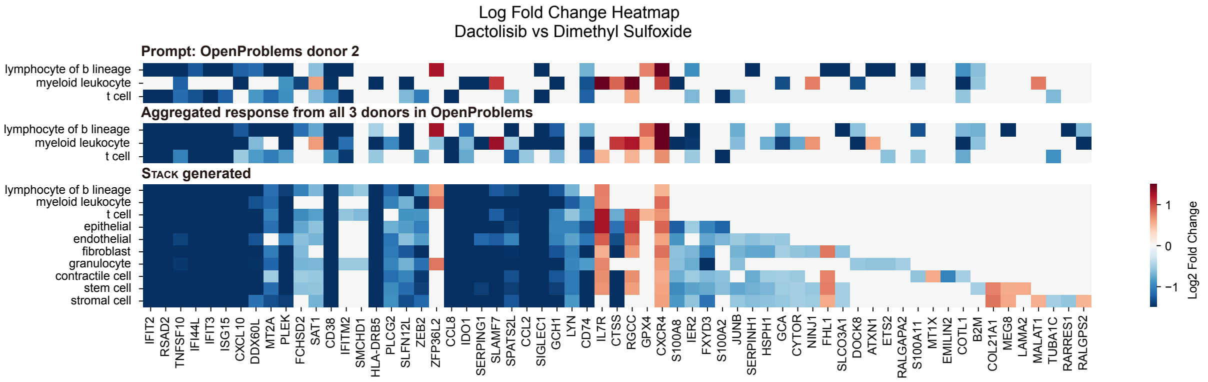

Figure S11 | Log2-fold-change heatmap of Dactolisib *Perturb Sapiens* versus control. Only significantly changed genes are shown in color, the rest are colored gray.

bioRxiv preprint doi: https://doi.org/10.64898/2026.01.09.698608; this version posted January 9, 2026. The copyright holder for this preprint (which was not certified by peer review) is the author/funder, who has granted bioRxiv a license to display the preprint in perpetuity. It is made available under aCC-BY 4.0 International license.

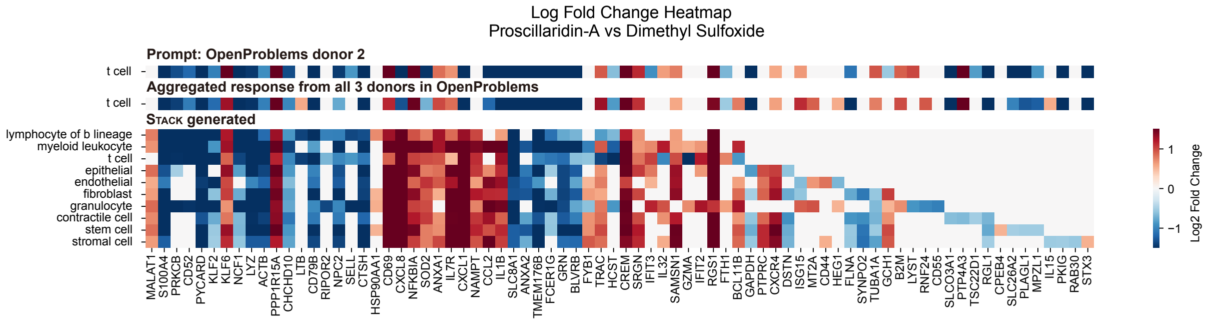

Figure S12 | Log2-fold-change heatmap of Proscillaridin-A *Perturb Sapiens* versus control. Only significantly changed genes are shown in color, the rest are colored gray.

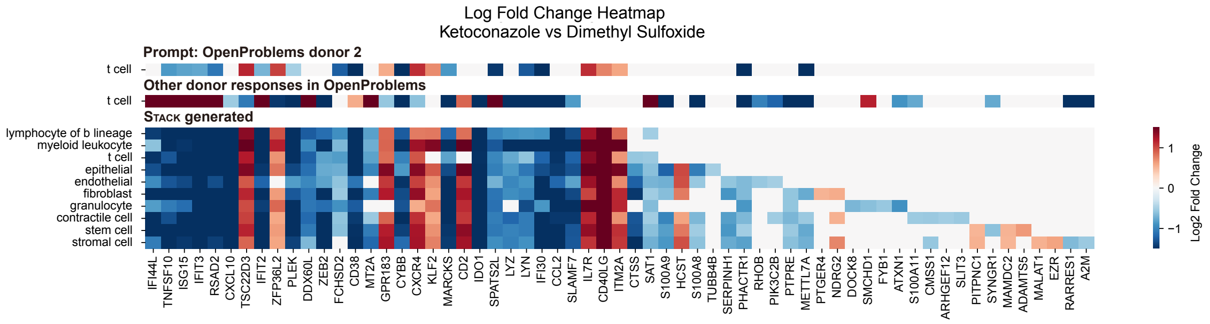

Figure S13 | Log2-fold-change heatmap of Ketoconazole *Perturb Sapiens* versus control. Only significantly changed genes are shown in color, the rest are colored gray.

bioRxiv preprint doi: https://doi.org/10.64898/2026.01.09.698608; this version posted January 9, 2026. The copyright holder for this preprint (which was not certified by peer review) is the author/funder, who has granted bioRxiv a license to display the preprint in perpetuity. It is made available under aCC-BY 4.0 International license.

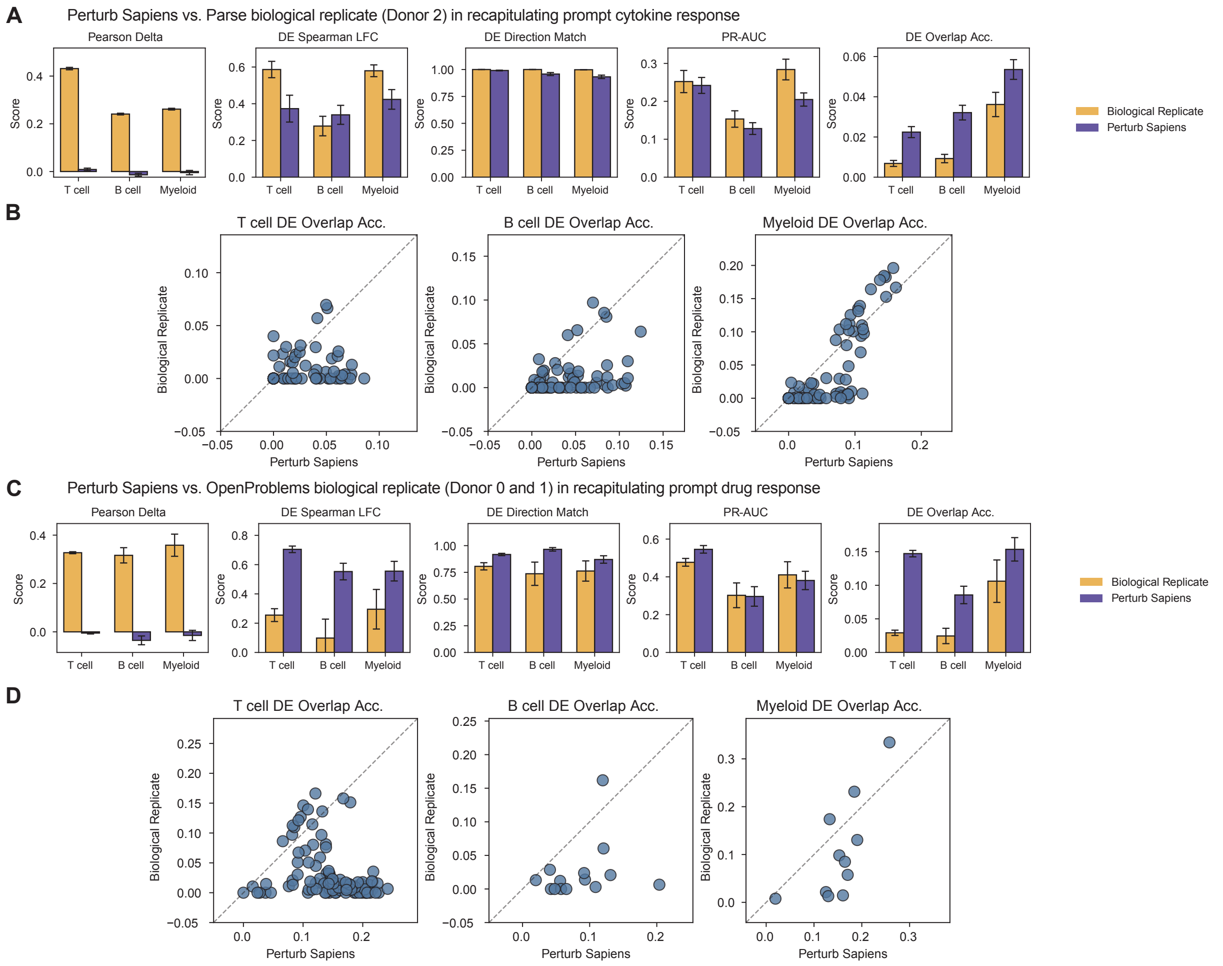

Figure S14 | Comparison of *Perturb Sapiens* immune cells with biological replicates. **A.** Evaluation on Parse cytokine perturbations using Donor 2 as the biological replicate. **B.** Scatter plot of differential expression (DE) overlap accuracy between *Perturb Sapiens* and the biological replicate for each cytokine (n=90). **C.** Evaluation on OpenProblems drug perturbations using both remaining donors as biological replicates due to limited cell numbers per individual donor. **D.** Scatter plot of DE overlap accuracy between *Perturb Sapiens* and biological replicates for each drug (n=111).

bioRxiv preprint doi: https://doi.org/10.64898/2026.01.09.698608; this version posted January 9, 2026. The copyright holder for this preprint (which was not certified by peer review) is the author/funder, who has granted bioRxiv a license to display the preprint in perpetuity. It is made available under aCC-BY 4.0 International license.

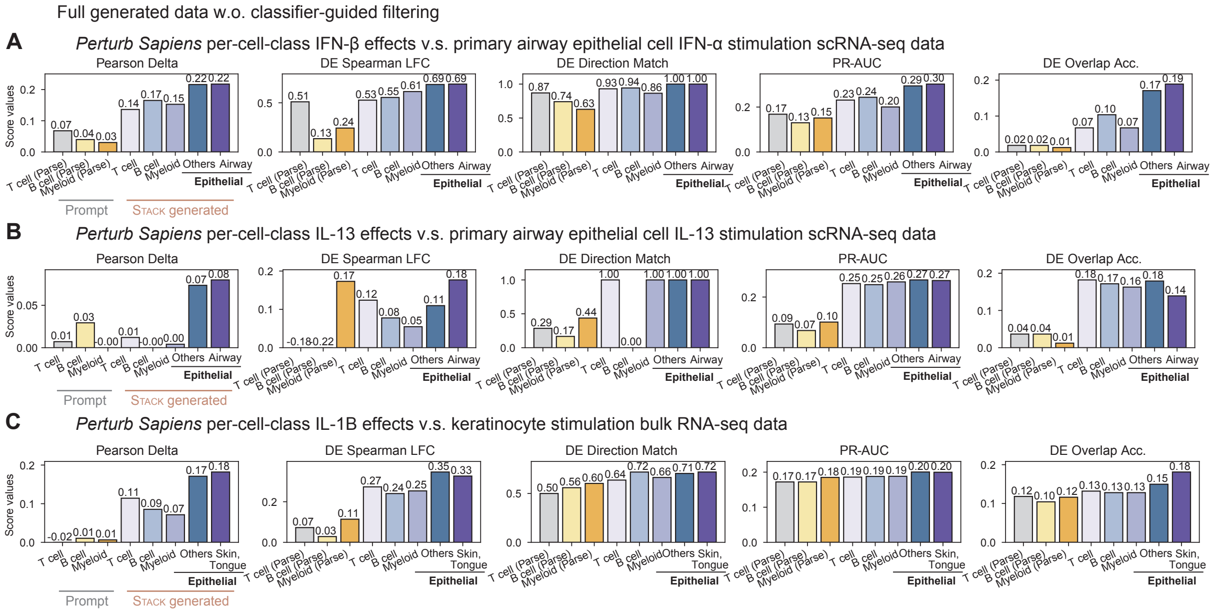

Figure S15 | Results of *Perturb Sapiens* without classifier-guided filtering. **A.** Evaluation of *Perturb Sapiens* epithelial interferon-beta (IFN-β) effects using single-cell IFN-α stimulation data from primary airway epithelial cells (Koh et al., 2023). **B.** Evaluation of *Perturb Sapiens* epithelial interleukin-13 (IL-13) effects using single-cell IL-13 stimulation data from primary airway epithelial cells (Koh et al., 2023). **C.** Evaluation of *Perturb Sapiens* epithelial interleukin-1 beta (IL-1β) effects using bulk IL-1β stimulation data from primary keratinocytes (Swindell et al., 2018).

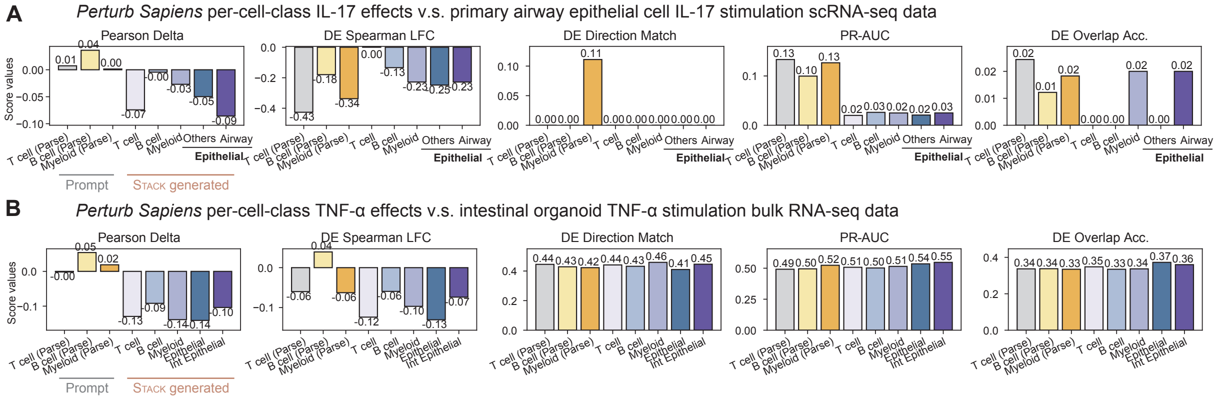

Figure S16 | Results of *Perturb Sapiens* on additional cytokines. **A.** Evaluation of *Perturb Sapiens* epithelial IL-17 effects using single-cell IL-17 stimulation data from primary airway epithelial cells (Koh et al., 2023). **B.** Evaluation of *Perturb Sapiens* epithelial TNF-α effects using bulk TNF-α stimulation data from primary intestinal epithelial cells (Lee et al., 2022).

bioRxiv preprint doi: https://doi.org/10.64898/2026.01.09.698608; this version posted January 9, 2026. The copyright holder for this preprint (which was not certified by peer review) is the author/funder, who has granted bioRxiv a license to display the preprint in perpetuity. It is made available under aCC-BY 4.0 International license.

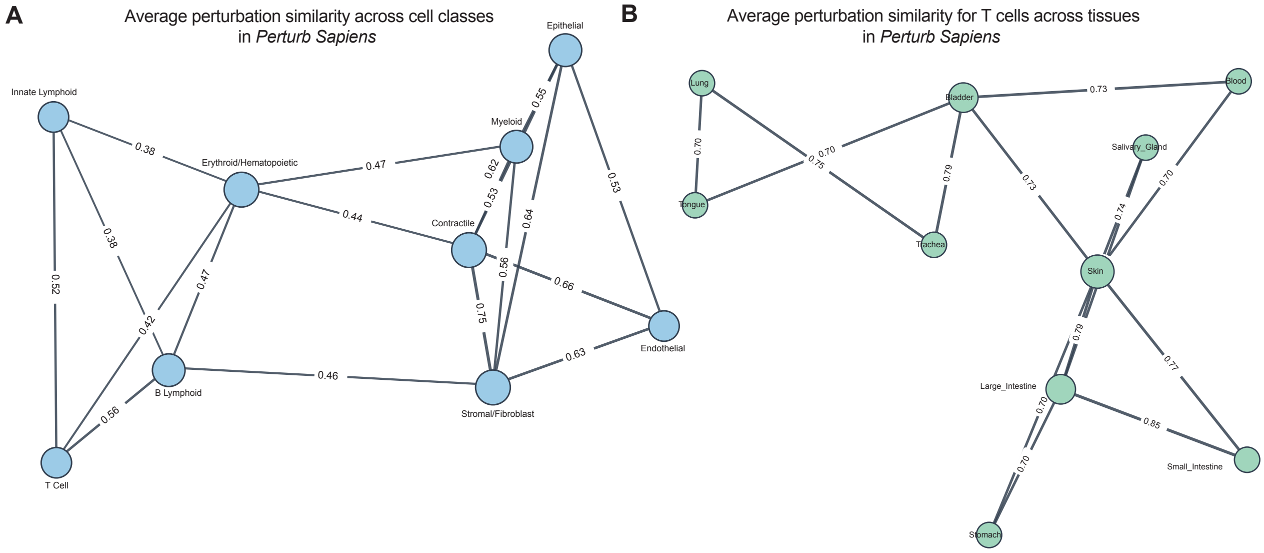

Figure S17 | Global characterization of *Perturb Sapiens*. **A.** Average perturbation similarity across cell classes in *Perturb Sapiens*. **B.** Average perturbation similarity for T cells across representative tissues in *Perturb Sapiens*. Values represent mean Spearman correlations averaged across perturbations using Fisher z-transformation. The top three or two edges by correlation are shown for each node.

bioRxiv preprint doi: https://doi.org/10.64898/2026.01.09.698608; this version posted January 9, 2026. The copyright holder for this preprint (which was not certified by peer review) is the author/funder, who has granted bioRxiv a license to display the preprint in perpetuity. It is made available under aCC-BY 4.0 International license.

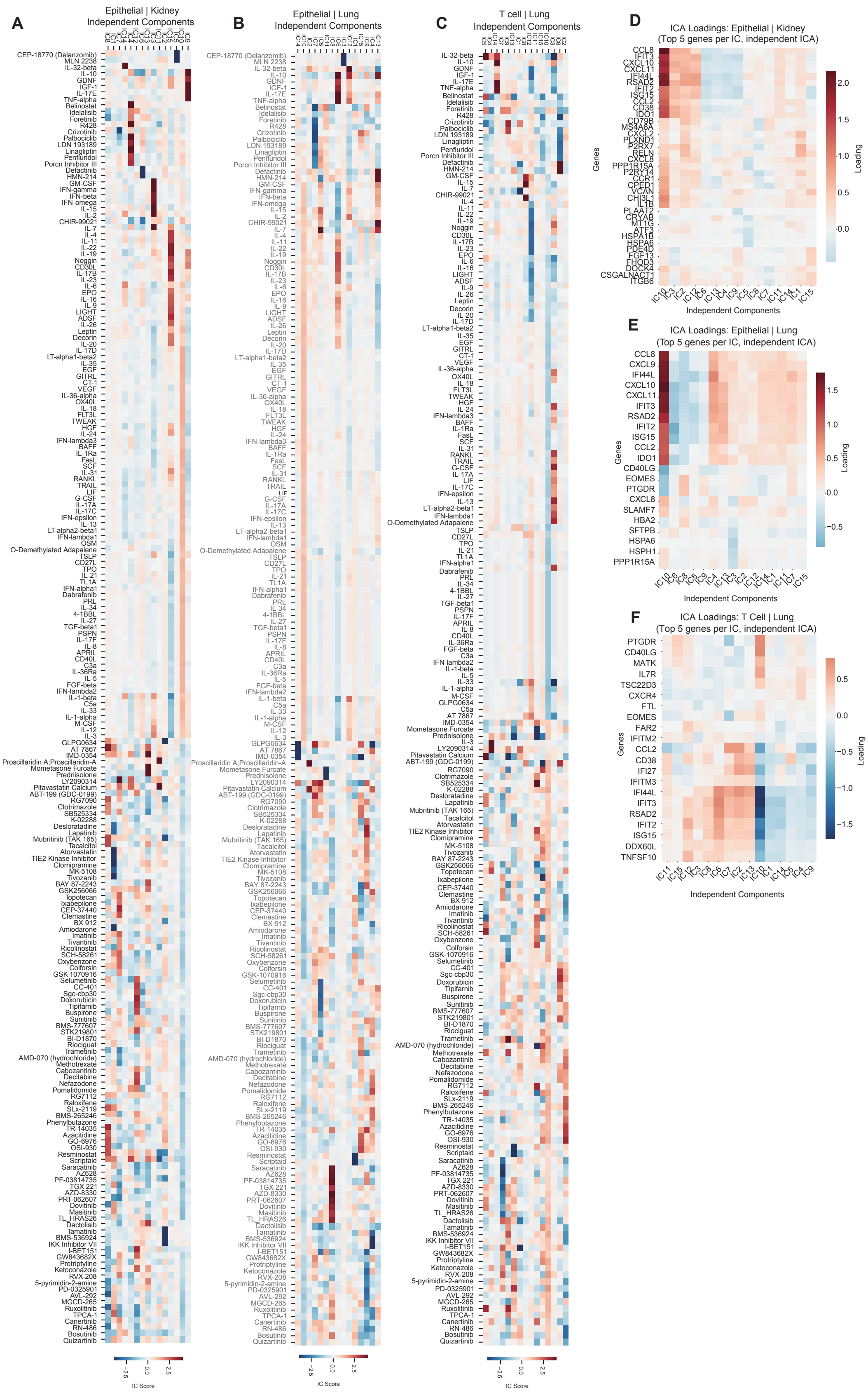

Figure S18 | Heatmap of independent components derived from *Perturb Sapiens* drug and cytokine log-fold changes (LFCs) in representative cell types and tissues. **A.** Epithelial Kidney. **B.** Epithelial lung. **C.** T cell lung. **D.** Factor loading for epithelial kidney. **E.** Factor loading for epithelial lung. **F.** Factor loading for T cell lung. All analyses used an independent component number of 15.

bioRxiv preprint doi: https://doi.org/10.64898/2026.01.09.698608; this version posted January 9, 2026. The copyright holder for this preprint (which was not certified by peer review) is the author/funder, who has granted bioRxiv a license to display the preprint in perpetuity. It is made available under aCC-BY 4.0 International license.

Table S1 | Additional evaluation of *Perturb Sapiens* intestinal epithelial cytokine responses against in vitro TNF-α bulk data (Saito et al., 2021). Significance: ** $p < 0.01$, *** $p < 0.001$, n.s. not significant.

Pearson Delta	DE Spearman LFC	DE Direction Match	PR AUC	DE Overlap Accuracy
-0.122^{***}	-0.077^{**}	0.434	0.530	0.267

Table S2 | Representative genes showing consistent (top) and opposite (bottom) trends between *Perturb Sapiens*, Parse T cells and in vitro TNF-α data (Lee et al., 2022).

Gene	*Perturb Sapiens* LFC	Adj p-value	Parse T cell LFC	Adj p-value	In vitro data LFC	Adj p-value
**Consistent trends between *Perturb Sapiens*/Parse and Lee et al. (2022)**						
MT1M	-1.257	$< 10^{-30}$	-2.281	1.99×10^{-3}	-4.115	6.69×10^{-5}
MT2A	-2.215	$< 10^{-30}$	-1.294	$< 10^{-30}$	-3.087	1.74×10^{-5}
MT1E	-0.900	$< 10^{-30}$	-1.114	3.66×10^{-3}	-3.360	3.90×10^{-4}
SOCS3	-0.463	$< 10^{-30}$	-1.218	$< 10^{-30}$	-2.388	1.88×10^{-5}
PLAU	0.314	2.69×10^{-10}	0.611	1.56×10^{-8}	1.815	2.86×10^{-8}
LDLR	-0.470	$< 10^{-30}$	-0.406	3.52×10^{-25}	-0.416	3.07×10^{-2}
B4GALT5	-0.515	$< 10^{-30}$	-0.501	$< 10^{-30}$	-0.458	2.95×10^{-4}
ITGAV	0.715	$< 10^{-30}$	0.425	1.00×10^{-9}	2.085	3.63×10^{-22}
MTSS1	0.850	$< 10^{-30}$	1.082	$< 10^{-30}$	1.825	2.27×10^{-2}
STOM	-0.582	$< 10^{-30}$	-1.231	$< 10^{-30}$	-1.784	7.39×10^{-6}
GSN	-1.001	$< 10^{-30}$	-0.536	$< 10^{-30}$	-0.962	1.98×10^{-5}
TGFA	0.868	$< 10^{-30}$	0.720	6.09×10^{-21}	0.753	1.01×10^{-4}
**Opposite trends between *Perturb Sapiens*/Parse and Lee et al. (2022)**						
NFKBIA	-0.499	$< 10^{-30}$	-0.727	$< 10^{-30}$	1.822	3.65×10^{-10}
SOD2	-0.429	$< 10^{-30}$	-1.131	$< 10^{-30}$	0.647	2.80×10^{-3}
RHOB	-0.269	5.11×10^{-9}	-0.769	3.26×10^{-13}	0.491	2.01×10^{-2}
SAT1	-0.952	$< 10^{-30}$	-0.651	$< 10^{-30}$	1.046	7.50×10^{-5}
GCH1	-1.288	$< 10^{-30}$	-1.618	$< 10^{-30}$	0.356	3.19×10^{-4}
RIPK2	-1.168	$< 10^{-30}$	-0.651	$< 10^{-30}$	1.065	2.83×10^{-6}
DRAM1	-0.631	$< 10^{-30}$	-1.316	$< 10^{-30}$	1.972	5.94×10^{-12}
ISG15	-3.814	$< 10^{-30}$	-1.729	$< 10^{-30}$	2.104	6.28×10^{-10}
IFIT2	-4.196	$< 10^{-30}$	-1.613	$< 10^{-30}$	2.081	2.79×10^{-20}
IFIT3	-3.947	$< 10^{-30}$	-1.506	$< 10^{-30}$	2.588	4.02×10^{-25}
RSAD2	-3.591	$< 10^{-30}$	-1.922	$< 10^{-30}$	3.273	3.34×10^{-8}
IFITM3	-1.010	$< 10^{-30}$	-1.540	$< 10^{-30}$	1.379	2.60×10^{-6}
IFI27	-1.439	$< 10^{-30}$	-2.269	$< 10^{-30}$	2.529	1.24×10^{-4}
CXCL10	-3.706	$< 10^{-30}$	-2.775	$< 10^{-30}$	6.025	8.72×10^{-21}
CXCL11	-3.528	$< 10^{-30}$	-2.938	$< 10^{-30}$	3.069	9.81×10^{-25}
TNFSF10	-3.015	$< 10^{-30}$	-1.674	$< 10^{-30}$	2.432	1.06×10^{-30}
CD74	-1.242	$< 10^{-30}$	-0.329	$< 10^{-30}$	3.979	$< 10^{-30}$
